# Cardiometabolic health–frailty phenotypes and bidirectional transitions between atrial fibrillation and heart failure: multistate trajectories and proteomic signatures

**DOI:** 10.1016/j.jnha.2026.101006

**Published:** 2026-10-10

**Authors:** Chuanyong Cui, Banghua Liu, Guangji Zhang, Zhongyan Du, Wenke Cheng

**Affiliations:** a Department of Cardiology, The First Affiliated Hospital of Bengbu Medical University, Bengbu, 233000, China; b Zhejiang Key Laboratory of Blood-Stasis-Toxin Syndrome, Zhejiang Chinese Medical University, Hangzhou, 310053, China; c School of Basic Medical Sciences, Zhejiang Chinese Medical University, Hangzhou, 310053, China; d Zhejiang Engineering Research Center for "Preventive Treatment" Smart Health of Traditional Chinese Medicine, Zhejiang Chinese Medical University, Hangzhou, 310053, China; e School of Life Science, Anhui Provincial Key Laboratory of Tumor Evolution and Intelligent Diagnosis and Treatment, Anhui Province Key Laboratory of Immunology in Chronic Diseases, Bengbu Medical University, Bengbu 233030, China

**Keywords:** Cardiometabolic health, Frailty, Atrial fibrillation, Heart failure, Proteomics

## Abstract

**Objective:**

To examine whether a joint cardiometabolic health–frailty phenotype is associated with incident atrial fibrillation (AF), incident heart failure (HF) and progression to AF–HF comorbidity, and to identify candidate circulating protein signals related to this phenotype.

**Methods:**

Among 319,048 UK Biobank participants without AF or HF, cardiometabolic status (healthy [CMH] or unhealthy [CMU]) and Frailty status (non-frail [NF] or pre-frail/frail [F]) defined four phenotypes. Cox and clock-forward multistate models estimated disease-specific and transition-specific hazards. Proteomic feature selection and exploratory mediation analyses included 28,206 participants with Olink plasma proteomic data.

**Results:**

During follow-up, 17,844 participants developed AF, 5,410 developed HF, and 2,403 and 791 progressed from AF and HF, respectively, to comorbidity. Compared with CMH-NF, CMU-F was associated with the greatest risks of incident HF (hazard ratio (HR), 2.76; 95% confidence interval (CI), 2.59–2.95) and AF (HR, 1.49; 95% CI, 1.43–1.55). Corresponding multistate HRs were 2.98 (95% CI, 2.76–3.22) for baseline-to-HF, 1.45 (1.39–1.51) for baseline-to-AF, 1.76 (1.57–1.98) for AF-to-comorbidity, and 1.27 (1.02–1.57) for HF-to-comorbidity. Positive additive interactions indicated excess risk from coexisting cardiometabolic unhealthiness and frailty. Thirteen proteins showed statistical mediation signals for both outcomes; FABP4 yielded the largest positive mediated proportions (AF, 49.8%; HF, 32.9%) and transition-specific signals for baseline-to-AF and AF-to-comorbidity.

**Conclusions:**

Coexisting cardiometabolic unhealthiness and frailty identified individuals at greatest risk of AF, HF, and subsequent comorbidity. Exploratory proteomic analyses identified FABP4 as the most consistent preliminary signal, which warrants further validation in independent cohorts.

## 1 Introduction

Atrial fibrillation (AF) and heart failure (HF) frequently coexist and are linked by bidirectional pathophysiological mechanisms. AF may promote HF through rapid ventricular activation, loss of atrial contraction, and impaired ventricular filling [[1], [2], [3]], whereas HF predisposes to AF through atrial pressure–volume overload, fibrosis, and electrical remodelling [4,5]. A key unresolved question is which upstream phenotypes identify individuals most likely to enter and progress through the AF–HF disease trajectory. Cardiometabolic unhealthiness and frailty capture complementary dimensions of vulnerability—cumulative metabolic–vascular injury and diminished multisystem reserve, respectively [6,7]. Although both have been associated with AF and HF, previous studies have generally considered these conditions as isolated endpoints [[8], [9], [10]]. Whether coexisting cardiometabolic unhealthiness and frailty identify individuals at disproportionate risk of first-onset AF or HF and subsequent progression to AF–HF comorbidity, and whether their joint effect exceeds that associated with either condition alone, remain unclear.

Large-scale plasma proteomics may further delineate molecular signatures linking complex clinical phenotypes to cardiovascular disease [11]. Using prospective UK Biobank data, we therefore examined associations of joint cardiometabolic health–frailty phenotypes with incident AF, incident HF, and transitions from either condition to AF–HF comorbidity. We additionally integrated Olink proteomics to identify phenotype-associated proteins and characterize circulating proteomic patterns associated with these phenotypes and transitions.

## 2 Methods

### 2.1 Study design and participants

The UK Biobank recruited >500,000 adults aged 40–69 years at 22 centres across the United Kingdom during 2006–2010 [12]. Participants provided written informed consent, and ethical approval was obtained from the North West Multi-centre Research Ethics Committee (11/NW/0382). The study was registered with the Medical Research Registration Information System (registration no. MR-34-26-058122). After the data screening process, 319,048 participants remained (Figure S1). Among them, 28,206 had Olink plasma proteomic data.

### 2.2 Cardiometabolic health–frailty phenotypes

Five cardiometabolic abnormalities were assessed: adiposity; dysglycaemia; elevated blood pressure; hypertriglyceridaemia; and low high-density lipoprotein cholesterol [13]. Cardiometabolic unhealthiness was defined as ≥3 abnormalities. Frailty was assessed using the Fried phenotype (Table S2) [14]. Participants meeting 0, 1–2, or ≥3 criteria were classified as robust, pre-frail, or frail, respectively. Pre-frail and frail participants were combined in the primary analysis, defining four joint phenotypes: cardiometabolically healthy and non-frail (CMH-NF), cardiometabolically healthy and pre-frail/frail (CMH-F), cardiometabolically unhealthy and non-frail (CMU-NF), and cardiometabolically unhealthy and pre-frail/frail (CMU-F). Detailed information is provided in Supplementary Methods.

### 2.3 Ascertainment of outcomes

Incident AF and HF were identified from linked primary-care, hospital, death-registry, and self-reported records using ICD-10 codes I48 and I50 (Table S3) [15,16], respectively. AF–HF comorbidity was dated at the second diagnosis. Four transition pathways were assessed according to the temporal sequence of disease onset: baseline to AF, baseline to HF, AF to comorbidity and HF to comorbidity. Detailed information is provided in Supplementary Methods.

### 2.4 Proteomic data

Plasma proteomic data were obtained from the UK Biobank Pharma Proteomics Project, which quantified 2,923 proteins using Olink Explore and reported abundance as log2-normalized protein expression [17]. Analyses included 28,206 eligible participants. Proteins with >20% missingness or zero variance were excluded; missing values among the remaining 2,911 proteins were K-nearest-neighbour imputed, and abundances were standardized to z-scores.

### 2.5 Covariates

Covariates were selected a priori based on prior epidemiological evidence and their potential associations with cardiometabolic health, frailty and cardiovascular outcomes [18,19]. Multivariable models included age, sex, race, smoking status, drinking status, socioeconomic deprivation, diet quality (Table S4) and kidney function. Continuous covariates had <3% missingness and were imputed using mean values. Missing values for categorical covariates were retained as separate unknown categories to preserve the analytical sample size. Detailed information is provided in Supplementary Methods.

### 2.6 Statistical analysis

Continuous variables are presented as mean ± standard deviation, and categorical variables as counts and percentages. Cox proportional hazards models estimated hazard ratios (HRs) and 95% confidence intervals (CIs) for incident AF and HF. Clock-forward multistate models evaluated four transitions. CMH-NF was the reference group. Proportional-hazards assumptions were assessed using Schoenfeld residuals.

Model-adjusted cumulative transition probability curves were generated to depict disease progression across Cardiometabolic Health–Frailty Phenotype categories throughout follow-up. Additive interaction was assessed using the relative excess risk due to interaction (RERI), attributable proportion (AP) and synergy index; multiplicative interaction was evaluated by likelihood-ratio testing. Subgroup analyses were prespecified by age and sex. Sensitivity analyses examined early-event exclusion, using multiple imputation for missing covariates, restriction to White participants, alternative cardiometabolic thresholds, additional biochemical adjustment, latent-class phenotypes, separate pre-frailty and frailty effects, re-inclusion of baseline cancer or cardiovascular disease, and multistate models incorporating death as an absorbing state.

Proteome-wide association analyses (PWAS) examined cardiometabolic status and frailty separately. Bonferroni-significant proteins underwent pathway enrichment and elastic-net selection; proteins consistently selected across all four mixing parameters and shared by both exposure domains defined the candidate signature. Exploratory mediation analyses evaluated individual proteins as statistical mediators of CMU-F versus CMH-NF associations with incident AF, HF, and multistate transitions. The total natural indirect effect was primary, with mediated proportions interpreted descriptively. Benjamini–Hochberg correction controlled the false discovery rate. Because phenotypes and proteins were measured concurrently, mediation estimates were regarded as statistical decompositions rather than causal effects. Analyses used R version 4.2.3, with two-sided tests. Detailed statistical analyses procedures are provided in Supplementary Methods.

## 3 Results

### 3.1 Study population and cardiovascular transitions

Among 319,048 participants (mean age, 55.8 years; 54.1% women), 5,410 developed HF, 17,844 developed AF, and 791 and 2,403 subsequently progressed from HF and AF, respectively, to AF–HF comorbidity during a median 15.7-year follow-up (Table 1; Figure S2). Participants undergoing cardiovascular transitions had less favourable clinical profiles, with the greatest cardiometabolic and frailty burden observed among those progressing from HF to comorbidity.

**Table 1:** Baseline characteristics of the study population across multi-state transition groups.

Variable	Total (N = 319,048)	Baseline to HF (N = 5,410)	Baseline to AF (N = 17,844)	HF to Comorbidity (N = 791)	AF to Comorbidity (N = 2,403)
Age (years)	55.81 (8.07)	60.63 (6.87)	61.15 (6.35)	62.12 (6.1)	62.72 (5.46)
Sex					
Female	172733 (54.1%)	2212 (40.9%)	6998 (39.2%)	266 (33.6%)	902 (37.5%)
Male	146315 (45.9%)	3198 (59.1%)	10846 (60.8%)	525 (66.4%)	1501 (62.5%)
Race					
White	302539 (94.8%)	5122 (94.7%)	17409 (97.6%)	753 (95.2%)	2331 (97%)
Black	4304 (1.3%)	76 (1.4%)	85 (0.5%)	8 (1%)	16 (0.7%)
Asian	6673 (2.1%)	139 (2.6%)	163 (0.9%)	21 (2.7%)	30 (1.2%)
Other	5532 (1.7%)	73 (1.3%)	187 (1%)	9 (1.1%)	26 (1.1%)
Smoking status					
Never	178450 (55.9%)	2318 (42.8%)	8512 (47.7%)	334 (42.2%)	1018 (42.4%)
Previous	32940 (10.3%)	960 (17.7%)	1847 (10.4%)	126 (15.9%)	299 (12.4%)
Current	106751 (33.5%)	2106 (38.9%)	7408 (41.5%)	326 (41.2%)	1075 (44.7%)
Missing	907 (0.3%)	26 (0.5%)	77 (0.4%)	5 (0.6%)	11 (0.5%)
Drinking status					
Never	12668 (4%)	299 (5.5%)	621 (3.5%)	46 (5.8%)	89 (3.7%)
Previous	10187 (3.2%)	321 (5.9%)	679 (3.8%)	49 (6.2%)	109 (4.5%)
Current	295963 (92.8%)	4784 (88.4%)	16525 (92.6%)	696 (88%)	2201 (91.6%)
Missing	230 (0.1%)	6 (0.1%)	19 (0.1%)	0 (0%)	4 (0.2%)
Dietary score	5.07 (1.53)	5.28 (1.51)	5.09 (1.52)	5.31 (1.47)	5.16 (1.52)
TDI	−1.41 (3.02)	−0.78 (3.31)	−1.41 (3.02)	−0.87 (3.26)	−1.08 (3.13)
eGFR (mL/min/1.73 m²)	95.36 (12.79)	91.61 (14.89)	91.64 (13.23)	89.97 (16.53)	90.04 (14)
AST (U/L)	23.54 (14.12)	25.25 (15.46)	24.23 (13.85)	25.51 (15.98)	24.59 (14.22)
ALT (U/L)	26.07 (10.43)	27.73 (13.51)	27.12 (11.53)	27.85 (11.74)	27.78 (11.53)
CRP (mg/L)	2.49 (4.12)	3.89 (5.8)	3.01 (4.77)	4.1 (6.07)	3.81 (5.61)
Number of cardiometabolic abnormalities					
any 0 cardiometabolic abnormalities	29786 (9.3%)	124 (2.3%)	692 (3.9%)	15 (1.9%)	36 (1.5%)
any 1 cardiometabolic abnormalities	63185 (19.8%)	508 (9.4%)	2426 (13.6%)	54 (6.8%)	235 (9.8%)
any 2 cardiometabolic abnormalities	83970 (26.3%)	1123 (20.8%)	4514 (25.3%)	147 (18.6%)	551 (22.9%)
any 3 cardiometabolic abnormalities	70324 (22%)	1285 (23.8%)	4195 (23.5%)	189 (23.9%)	575 (23.9%)
any 4 cardiometabolic abnormalities	49082 (15.4%)	1335 (24.7%)	3780 (21.2%)	211 (26.7%)	535 (22.3%)
any 5 cardiometabolic abnormalities	22701 (7.1%)	1035 (19.1%)	2237 (12.5%)	175 (22.1%)	471 (19.6%)
Frailty states					
Frail	8749 (2.7%)	497 (9.2%)	768 (4.3%)	84 (10.6%)	168 (7%)
Pre-frail	116146 (36.4%)	2502 (46.2%)	7047 (39.5%)	355 (44.9%)	1089 (45.3%)
Robust	194153 (60.9%)	2411 (44.6%)	10029 (56.2%)	352 (44.5%)	1146 (47.7%)
Cardiometabolic Health–Frailty Phenotype					
CMH-NF	116436 (36.5%)	943 (17.4%)	4780 (26.8%)	120 (15.2%)	454 (18.9%)
CMH-F	60505 (19%)	812 (15%)	2852 (16%)	96 (12.1%)	368 (15.3%)
CMU-NF	77717 (24.4%)	1468 (27.1%)	5249 (29.4%)	232 (29.3%)	692 (28.8%)
CMU-F	64390 (20.2%)	2187 (40.4%)	4963 (27.8%)	343 (43.4%)	889 (37%)

TDI, Townsend deprivation index; eGFR, estimated glomerular filtration rate; ALT, alanine aminotransferase; AST, aspartate aminotransferase; CRP, C-reactive protein; CMH-NF,cardiometabolically healthy and non-frail; CMH-F, cardiometabolically healthy and pre-frail/frail; CMU-NF, cardiometabolically unhealthy and non-frail; CMU-F, cardiometabolically unhealthy and pre-frail/frail.

### 3.2 Multistate model analysis, interactions, and cumulative transition probabilities

Compared with CMH-NF, all adverse phenotypes were associated with incident HF [adjusted HRs (95% CIs): CMH-F, 1.60 (1.48–1.73); CMU-NF, 1.58 (1.48–1.69); CMU-F, 2.76 (2.59–2.95)] and AF [1.21 (1.16–1.27), 1.17 (1.12–1.22), and 1.49 (1.43–1.55), respectively] (Table 2). In multistate models, CMU-F showed the strongest associations across transitions: baseline-to-HF, 2.98 (2.76–3.22); baseline-to-AF, 1.45 (1.39–1.51); AF-to-comorbidity, 1.76 (1.57–1.98); and HF-to-comorbidity, 1.27 (1.02–1.57). CMH-F and CMU-NF were also associated with both baseline transitions and AF-to-comorbidity, but not HF-to-comorbidity.

**Table 2:** Associations of Cardiometabolic Health–Frailty Phenotypes with dynamic transitions between heart failure and atrial fibrillation, evaluated using conventional Cox regression and multistate models.

	N	Events	Proportion (%)	HR (95% CI)	P value	P for FDR
**Traditional Cox model**						
**HF**						
CMH-F	60505	1180	2	1.60 (1.48, 1.73)	<0.001	
CMU-NF	77717	2160	2.8	1.58 (1.48, 1.69)	<0.001	
CMU-F	64390	3076	4.8	2.76 (2.59, 2.95)	<0.001	
**AF**						
CMH-F	60505	2948	4.9	1.21 (1.16, 1.27)	<0.001	
CMU-NF	77717	5481	7.1	1.17 (1.12, 1.22)	<0.001	
CMU-F	64390	5306	8.2	1.49 (1.43, 1.55)	<0.001	
**Multi-state model**						
**Baseline to HF**						
CMH-F	60505	812	1.3	1.61 (1.47, 1.77)	<0.001	<0.001
CMU-NF	77717	1468	1.9	1.65 (1.52, 1.79)	<0.001	<0.001
CMU-F	64390	2187	3.4	2.98 (2.76, 3.22)	<0.001	<0.001
**Baseline to AF**						
CMH-F	60505	2852	4.7	1.20 (1.15, 1.26)	<0.001	<0.001
CMU-NF	77717	5249	6.8	1.16 (1.11, 1.20)	<0.001	<0.001
CMU-F	64390	4963	7.7	1.45 (1.39, 1.51)	<0.001	<0.001
**HF to Comorbidity**						
CMH-F	812	96	11.8	1.01 (0.77, 1.33)	0.925	0.925
CMU-NF	1468	232	15.8	1.13 (0.91, 1.42)	0.269	0.294
CMU-F	2187	343	15.7	1.27 (1.02, 1.57)	0.030	0.035
**AF to Comorbidity**						
CMH-F	2852	368	12.9	1.37 (1.20, 1.58)	<0.001	<0.001
CMU-NF	5249	692	13.2	1.31 (1.16, 1.48)	<0.001	<0.001
CMU-F	4963	889	17.9	1.76 (1.57, 1.98)	<0.001	<0.001

Traditional Cox models estimate associations for incident HF and AF, respectively. Multistate models evaluate transition-specific hazards, including transitions from baseline to HF, baseline to AF, and subsequent transitions to HF–AF comorbidity. Models were adjusted for age, sex, race, smoking status, drinking status, dietary score, TDI, eGFR.Nominal P values are reported alongside P for FDR-adjusted P values, which were calculated using the Benjamini–Hochberg. HF, heart failure; AF, atrial fibrillation; CMH-NF, cardiometabolically healthy and non-frail; CMH-F, cardiometabolically healthy and pre-frail/frail; CMU-NF, cardiometabolically unhealthy and non-frail; CMU-F, cardiometabolically unhealthy and pre-frail/frail; TDI, Townsend deprivation index; eGFR, estimated glomerular filtration rate.

No multiplicative interactions were detected. Positive additive interactions between cardiometabolic health and frailty were observed for incident HF and AF (RERI, 0.58 and 0.11, respectively) and their corresponding baseline transitions (0.71 and 0.09), but not for progression to comorbidity (Table S5). Model-adjusted cumulative transition probabilities differed across cardiometabolic health–frailty phenotypes (Figure S3 and Table S6). Across transition pathways, CMU-F consistently showed the highest probabilities, whereas CMH-NF showed the lowest.

### 3.3 Subgroup analysis and sensitivity results

Associations were stronger among participants aged <65 years and among women, with evidence of effect modification by age and sex. For progression to comorbidity, no clear demographic heterogeneity was observed (Figure S4). Sensitivity analyses broadly supported the robustness of the main findings (Figure S5 and Tables S7–S18).

### 3.4 Proteomic signatures and mediation analyses

Among 28,206 participants (Table S19), 2,071 of 2,911 proteins were associated with cardiometabolic unhealthiness and 811 with frailty. Both profiles were enriched for immune-inflammatory, cytokine-signalling, cell-adhesion, extracellular-matrix, and PI3K–Akt pathways. Elastic-net analyses identified 59 proteins shared between the two phenotypes (Figure S6, Supplementary Data 1–7).

Of these, 20 and 28 showed evidence of statistical mediation of CMU-F associations with incident AF and HF, respectively, including 13 shared candidates (Fig. 1). FABP4 showed the largest positive mediated proportions for AF (49.8%) and HF (32.9%), whereas ADIPOQ showed inverse mediation (−47.2% and −14.3%, respectively). FABP4 also showed statistical mediation of baseline-to-AF and AF-to-comorbidity transitions (56.4% and 59.7%, respectively) and remained consistently identified across alternative imputation and complete-case analyses (Tables S20–S25).

**Fig. 1:**
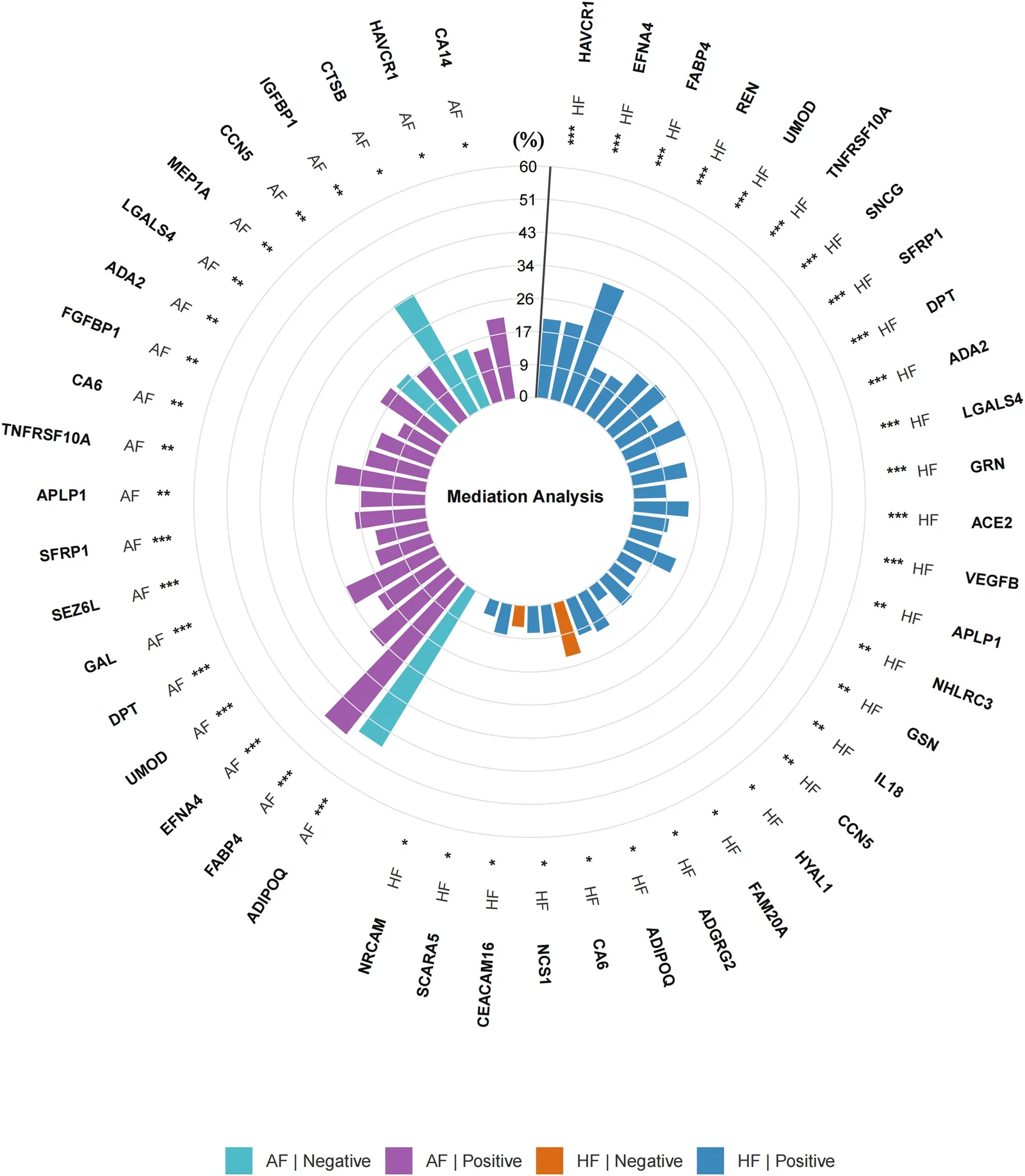
Estimated proportions mediated by circulating proteins in the associations of CMU-F phenotype with incident AF and HF. The circular bar plot shows the estimated proportion mediated by each protein for the associations of the CMU-F, compared with the CMH-NF, with incident AF and HF. Mediation proportions are expressed as percentages. Colours denote outcome and direction of the estimated mediation proportion: cyan, negative mediation for AF; purple, positive mediation for AF; orange, negative mediation for HF; and blue, positive mediation for HF. Mediation models were adjusted for age, sex, race, smoking status, drinking status, TDI, dietary score, and eGFR. Two-sided *P* values were adjusted using the FDR procedure. *0.01 ≤ FDR-adjusted *P* value <0.05; **0.001 ≤ FDR-adjusted *P* value <0.01; ***FDR-adjusted *P* value <0.001. ACE2, angiotensin-converting enzyme 2; ADA2, adenosine deaminase 2; ADGRG2, adhesion G protein-coupled receptor G2; ADIPOQ, adiponectin; APLP1, amyloid beta precursor-like protein 1; CA6, carbonic anhydrase 6; CA14, carbonic anhydrase 14; CCN5, cellular communication network factor 5; CEACAM16, carcinoembryonic antigen-related cell adhesion molecule 16; CTSB, cathepsin B; DPT, dermatopontin; EFNA4, ephrin-A4; FABP4, fatty acid-binding protein 4; FDR, false discovery rate; FGFBP1, fibroblast growth factor-binding protein 1; GAL, galanin; GRN, granulin precursor; GSN, gelsolin; HAVCR1, hepatitis A virus cellular receptor 1; HYAL1, hyaluronidase 1; IGFBP1, insulin-like growth factor-binding protein 1; IL18, interleukin-18; LGALS4, galectin-4; MEP1A, meprin A subunit alpha; NHLRC3, NHL repeat-containing protein 3; NRCAM, neuronal cell adhesion molecule; REN, renin; **FAM20A:** FAM20A Golgi-associated secretory pathway pseudokinase; **NCS1:** neuronal calcium sensor 1; SCARA5, scavenger receptor class A member 5; SEZ6L, seizure protein 6-like; SFRP1, secreted frizzled-related protein 1; SNCG, gamma-synuclein; TNFRSF10A, tumour necrosis factor receptor superfamily member 10A; UMOD, uromodulin; VEGFB, vascular endothelial growth factor B. AF, atrial fibrillation; HF, heart failure; CMH-NF, cardiometabolically healthy and non-frail; CMU-F, cardiometabolically unhealthy and pre-frail/frail; TDI, Townsend deprivation index; eGFR, estimated glomerular filtration rate.

## 4 Discussion

In this prospective cohort, the coexistence of cardiometabolic unhealthiness and frailty identified participants at greatest risk of incident HF, incident AF, and progression to AF–HF comorbidity, consistently across Cox, multistate, and cumulative-probability analyses. Positive additive interactions suggested complementary contributions of metabolic–vascular injury and diminished physiological reserve. The associations were most pronounced for transitions from the baseline state to incident AF or HF, particularly for the baseline-to-HF transition. Exploratory proteomic mediation analyses identified 13 shared candidates; FABP4 showed the most consistent positive signal, whereas ADIPOQ showed inverse signals.

Previous studies have independently linked cardiometabolic dysfunction and frailty to AF and HF [10,20]. By integrating these complementary domains within a multistate framework, our study demonstrated a graded risk pattern: either cardiometabolic unhealthiness or frailty conferred intermediate risks, whereas their coexistence conferred the greatest risk of disease onset and progression. Associations were strongest for transitions culminating in HF, particularly baseline-to-HF and AF-to-comorbidity, consistent with HF representing a downstream manifestation of convergent metabolic, vascular, renal, inflammatory, and neurohormonal stress [21,22]. Conversely, the weaker, less precise association with HF-to-comorbidity suggests that, after HF onset, incident AF may be driven predominantly by established atrial pressure–volume overload, fibrosis, and electrical remodelling [23,24]. Treatment patterns, survivor selection, and fewer events may have further attenuated this association. Collectively, these findings indicate that cardiometabolic health and frailty provide complementary prognostic information, primarily identifying systemic vulnerability before AF or HF develops and particularly susceptibility to HF-related progression.

Proteomic analyses provided exploratory biological context for the association between CMU-F and AF–HF progression. FABP4 showed the most consistent positive statistical mediation signals across incident AF and HF and the baseline-to-AF and AF-to-comorbidity transitions. Its established involvement in adipose dysfunction, lipid handling, immunometabolic activation, atrial cardiomyopathy, and fibrosis supports its relevance as a molecular correlate of the joint phenotype [25,26]. Conversely, ADIPOQ showed inverse mediation signals. Given the adiponectin paradox in ageing, HF, kidney disease, and multimorbidity, these findings may reflect altered adipokine regulation, compensatory responses, or disease-related metabolic adaptation rather than straightforward protection [27]. Signals for HAVCR1 suggest subclinical cardiorenal vulnerability [28], whereas SFRP1, and CCN5 implicate extracellular-matrix organization and tissue remodelling [29,30]. Collectively, these exploratory associations identify protein signals linked to cardiometabolic health–frailty phenotypes and AF–HF outcomes; their biological relevance requires independent replication and mechanistic validation. However, observational mediation cannot establish causality, and these candidate proteins require validation in longitudinal proteomic, genetic, and experimental studies.

Several limitations merit consideration. Cardiometabolic health–frailty phenotypes were assessed only at baseline, precluding evaluation of longitudinal changes. Combining pre-frailty and frailty may have obscured severity-specific associations. Finally, proteomic analyses were exploratory and restricted to a smaller subset, limiting generalisability. The uncertain tissue origins of circulating proteins and susceptibility of observational mediation analyses to reverse causation and residual confounding preclude causal interpretation.

## 5 Conclusion

The CMU-F phenotype identified individuals at greatest risk of incident AF, HF, and subsequent AF–HF comorbidity, particularly HF onset. Exploratory proteomic analyses yielded 13 shared statistical signals, with FABP4 showing the most consistent pattern; these findings are preliminary and require independent replication.

## Author contributions

Conceptualization: WKC; Methodology: CYC and WKC; Formal analysis and investigation: CYC and BHL; Writing—original draft preparation: CYC, BHL and WKC; Writing—review and editing: CYC, BHL, GJZ, ZYD, and WKC; Resources: WKC; Supervision: ZYD and WKC. All authors contributed to subsequent revisions and approved the final version. All authors read and approved the final manuscript.

## Consent for publication

Not applicable.

## Ethics approval and consent to participate

The UK Biobank was established with ethical clearance from the North West Multi-Centre Research Ethics Committee (REC reference: 11/NW/0382). Written informed consent have been provided by all participants.

## Declaration of Generative AI and AI-assisted technologies in the writing process

Generative AI was not used to create or develop the scientific content of this manuscript. AI-assisted tools were used only for limited language editing during revision to improve readability and polish the text.

## Funding

None.

## Data availability

Data can be accessed from a public and open repository. Interested researchers can apply for access to the UK Biobank data at https://www.ukbiobank.ac.uk and https://biobank.ndph.ox.ac.uk/ukb.

## Data availability

Data can be accessed from a public and open repository. Interested researchers can apply for access to the UK Biobank data at https://www.ukbiobank.ac.uk and https://biobank.ndph.ox.ac.uk/ukb.

## Declaration of competing interest

The authors declare that they have no conflict of interest.

The authors declare that they have no known competing financial interests or personal relationships that could have appeared to influence the work reported in this paper.

## Footnotes

Appendix A Supplementary data associated with this article can be found, in the online version, at http://dx.doi.org/10.1016/j.jnha.2026.101006.
