# Supplementary material for: Cardiometabolic health–frailty phenotypes and bidirectional transitions between atrial fibrillation and heart failure: multistate trajectories and proteomic signatures
Source: J Nutr Health Aging. 2026 Oct 10;30(12):101006. doi: 10.1016/j.jnha.2026.101006 (PMC13677947; doi:10.1016/j.jnha.2026.101006)
Supplement: Supplementary file 1 [file mmc1.docx]

**Supplement**

**Cardiometabolic Health–Frailty Phenotypes and Bidirectional Transitions Between Atrial Fibrillation and Heart Failure: Multistate Trajectories and Proteomic Signatures**

Chuanyong Cui, Banghua Liu, Guangji Zhang, Zhongyan Du , Wenke Cheng

**Supplementary Materials**

**Supplementary method**

**Table S1.** Items used to ascertain prior medication use

**Table S2.** Frailty criteria

**Table S3.** Diagnostic codes for diseases

**Table S4.** The calculation method for cumulative dietary risk

**Table S5.** Additive and multiplicative interactions between cardiometabolic health and frailty for incident HF, incident AF, and AF–HF comorbidity progression

**Table S6.** Cumulative transition probability matrix according to cardiometabolic health-frailty phenotype

**Table S7.** Sensitivity Analysis I. Associations of cardiometabolic health–frailty phenotypes with incident heart failure, incident atrial fibrillation, and AF–HF disease transitions after re-including participants with cancer at baseline

**Table S8.** Sensitivity Analysis II. Associations of cardiometabolic health–frailty phenotypes with incident heart failure, incident atrial fibrillation, and AF–HF disease transitions after excluding events occurring within the first year of follow-up

**Table S9.** Sensitivity Analysis III. Associations of cardiometabolic health–frailty phenotypes with incident heart failure, incident atrial fibrillation, and AF–HF disease transitions after multiple imputation of missing covariates

**Table S10.** Sensitivity Analysis IV. Associations of cardiometabolic health–frailty phenotypes with incident heart failure, incident atrial fibrillation, and AF–HF disease transitions among White participants

**Table S11.** Sensitivity Analysis Ⅴ.Associations of cardiometabolic health–frailty phenotypes with incident heart failure, incident atrial fibrillation, and AF–HF disease transitions using ≥1 metabolic abnormality to define cardiometabolic unhealthiness

**Table S12.** Sensitivity Analysis Ⅵ. Associations of cardiometabolic health–frailty phenotypes with incident heart failure, incident atrial fibrillation, and AF–HF disease transitions using ≥2 metabolic abnormalities to define cardiometabolic unhealthiness

**Table S13**. Sensitivity Analysis Ⅶ. Associations of cardiometabolic health–frailty phenotypes with incident heart failure, incident atrial fibrillation, and AF–HF disease transitions after re-including participants with prevalent cardiovascular disease at baseline

**Table S14.** Sensitivity Analysis Ⅷ. Associations of cardiometabolic health–frailty phenotypes with incident heart failure, incident atrial fibrillation, and AF–HF disease transitions after additional adjustment for alanine aminotransferase, aspartate aminotransferase, and C-reactive protein

**Table S15.** Sensitivity Analysis Ⅸ. Associations of latent class–derived cardiometabolic–frailty phenotypes with incident heart failure, incident atrial fibrillation, and AF–HF disease transitions

**Table S16.** Sensitivity Analysis Ⅹ. Associations of cardiometabolic health–frailty phenotypes with incident heart failure, incident atrial fibrillation, and AF–HF disease transitions after excluding pre-frail participants

**Table S17.** Sensitivity Analysis ⅩⅠ. Associations of cardiometabolic health–frailty phenotypes with incident heart failure, incident atrial fibrillation, and AF–HF disease transitions after excluding frail participants

**Table S18.** Sensitivity Analysis ⅩⅡ. Associations of cardiometabolic health–frailty phenotypes with cardiovascular disease and mortality transitions in the death-inclusive multistate model

**Table S19.** Baseline Characteristics of the Proteomics Subcohort

**Table S20.** Cox regression–based mediation analysis of candidate proteomic features linking the cardiometabolic health–frailty phenotype to AF and HF

**Table S21.** Multistate mediation analysis of candidate proteomic features linking the cardiometabolic health–frailty phenotype to AF–HF Comorbidity progression

**Table S22.** Cox regression–based mediation analysis of candidate proteomic features linking the cardiometabolic health–frailty phenotype to AF and HF using mean-imputed proteomic data

**Table S23.** Multistate mediation analysis of candidate proteomic features linking the cardiometabolic health–frailty phenotype to AF–HF comorbidity progression using mean-imputed proteomic data

**Table S24.** Cox regression–based mediation analysis of candidate proteomic features linking the cardiometabolic health–frailty phenotype to AF and HF using complete case proteomic data

**Table S25.** Multistate mediation analysis of candidate proteomic features linking the cardiometabolic health–frailty phenotype to AF–HF comorbidity progression using complete case proteomic data

**Figure S1.** The study flow chart

**Figure S2.** AF–HF multistate disease transition framework

**Figure S3.** Model-adjusted cumulative transition probabilities for AF–HF disease progression according to cardiometabolic health–frailty phenotype

**Figure S4.** Subgroup associations of cardiometabolic health–frailty phenotypes with AF–HF transitions by age and sex

**Figure S5.** Model selection, phenotype distribution, and internal reproducibility of the three-class cardiometabolic–frailty solution

**Figure S6.** Plasma proteomic signatures associated with cardiometabolic health and frailty and pathway enrichment with feature selection stability

**Supplementary method**

**Assessment of Cardiometabolic Health–Frailty Phenotype**

Cardiometabolic health was defined according to five metabolic abnormalities reflecting adiposity, dysglycaemia, elevated blood pressure, hypertriglyceridaemia ,and low HDL cholesterol.^13^ Participants were classified as having a cardiometabolic abnormality if they met any of the following criteria: (1) body mass index ≥25 kg/m² or waist circumference >94 cm in men or >80 cm in women; (2) glycated haemoglobin A1c ≥39 mmol/mol, type 2 diabetes diagnosis, or use of glucose-lowering medication; (3) systolic/diastolic blood pressure ≥130/85 mmHg , hypertension diagnosis, or use of antihypertensive medication; (4) triglycerides ≥1.70 mmol/L or use of lipid-lowering medication; and (5) high-density lipoprotein cholesterol ≤1.0 mmol/L in men or ≤1.3 mmol/L in women, or use of lipid-lowering medication. Detailed information on medication use is provided in Table S1. Participants with ≥3 abnormalities were classified as cardiometabolically unhealthy; those with <3 abnormalities were classified as cardiometabolically healthy.

Physical frailty was assessed using the Fried frailty phenotype, which comprises unintentional weight loss, exhaustion, low grip strength, slow walking speed and low physical activity.^14^ These criteria were operationalised using UK Biobank touchscreen questionnaire data and physical measurements. Weight loss, exhaustion, walking speed and physical activity were derived from self-reported data. Grip strength was measured using a hydraulic dynamometer, and the higher value from either hand was used. Low grip strength was defined using sex- and body mass index-specific cut-off values. Participants were classified as non-frail, pre-frail or frail if they met 0, 1–2 or ≥3 criteria, respectively, consistent with previous UK Biobank applications of the Fried phenotype. Participants with missing data for any frailty component were excluded. Detailed criteria are provided in **Table S2**.

To construct the joint cardiometabolic health–frailty phenotype, frailty status was dichotomised as non-frail versus pre-frail/frail. This approach was used because pre-frailty reflects early impairment in physiological reserve and has been associated with adverse health outcomes. Participants were then categorized into four mutually exclusive groups: cardiometabolically healthy and non-frail (CMH-NF), cardiometabolically healthy and pre-frail/frail (CMH-F), cardiometabolically unhealthy and non-frail (CMU-NF), and cardiometabolically unhealthy and pre-frail/frail (CMU-F).

**Ascertainment of outcomes**

The primary outcomes were incident AF and incident HF. Outcomes were ascertained from the UK Biobank First Occurrences dataset (Category 1712), which integrates primary care, hospital inpatient, death registry and self-reported records, with diagnoses harmonised to three-character ICD-10 codes. Incident AF and HF were defined as the first recorded occurrence of ICD-10 codes I48 and I50, respectively.^15,16^ Diagnostic codes are listed in **Table S3**.

For each outcome, follow-up accrued from baseline until the first occurrence of the outcome of interest, death, loss to follow-up or administrative censoring, whichever occurred first. Incident AF and HF records were available through 1 July 2025, whereas death registry data were available through 2 December 2024. We therefore used 2 December 2024 as the administrative censoring date to ensure a consistent follow-up window across outcomes.

AF–HF comorbidity progression was evaluated using a transition-based framework among participants free of both AF and HF at baseline. Comorbidity was defined as the occurrence of both conditions during follow-up, with the second diagnosis marking entry into the comorbid state. Four transition pathways were assessed according to the temporal sequence of disease onset: baseline to AF, baseline to HF, AF to comorbidity and HF to comorbidity.

**Covariates**

Covariates were selected a priori based on prior epidemiological evidence and their potential associations with cardiometabolic health, frailty and cardiovascular outcomes.^18,19^ Multivariable models included age, sex, race, smoking status, drinking status, socioeconomic deprivation, diet quality and kidney function.

Baseline covariates were obtained from UK Biobank touchscreen questionnaires, nurse-led interviews, physical measurements and laboratory assays. Age, sex, race, smoking status and drinking status were derived from baseline questionnaires or interview data. Race was classified as White, Black, Asian or other according to self-reported information. Smoking status was categorized as never, previous, current or unknown, and alcohol consumption as never, former, current or unknown. Socioeconomic deprivation was assessed using the Townsend deprivation index (TDI), with higher values indicating greater deprivation. Diet quality was quantified using a dietary score reflecting overall dietary health (**Table S4**). Kidney function was assessed using estimated glomerular filtration rate (eGFR), calculated with the 2021 race-free CKD-EPI creatinine equation.

Continuous covariates had <3% missingness and were imputed using mean values. Missing values for categorical covariates were retained as separate unknown categories to preserve the analytical sample size.

**Statistical Analysis**

Continuous variables are presented as mean ± standard deviation, and categorical variables as counts and percentages. Cox proportional hazards models were used to estimate associations of the cardiometabolic health–frailty phenotype with incident AF and incident HF. The CMH-NF group was used as the reference. Hazard ratios (HRs) and 95% confidence intervals (CIs) were estimated for the CMH-F, CMU-NF and CMU-F groups. All models were adjusted for age, sex, race, smoking status, drinking status, TDI, dietary score and eGFR. Proportional hazards assumptions were verified via Schoenfeld residuals, with no substantial violations observed.

Disease progression from an AF/HF-free state to single disease and subsequent comorbidity was assessed using a clock-forward multistate framework implemented with the mstate package in R. Four transitions were specified: baseline to AF, baseline to HF, AF to comorbidity and HF to comorbidity. Participants with same-day onset of AF and HF were excluded because temporal sequence could not be determined. Model-adjusted cumulative transition probability curves were generated to depict disease progression across cardiometabolic health–frailty phenotype categories throughout follow-up. Transition probabilities at 1, 3, 5, 10, and 15 years were extracted for presentation.

Additive interaction between cardiometabolic health and frailty was assessed using the relative excess risk due to interaction (RERI), attributable proportion (AP) and synergy index, with 95% CIs estimated using the delta method. Multiplicative interaction was tested by adding a product term to the Cox model and comparing nested models using likelihood-ratio tests. Prespecified subgroup analyses were conducted by age (<65 versus ≥65 years) and sex, with heterogeneity assessed using interaction terms and likelihood-ratio tests.

Sensitivity analyses included: (1) re-including participants with cancer at baseline; (2) excluding events occurring within the first year of follow-up; (3) using multiple imputation for missing covariates; (4) restricting the analysis to White participants; (5) applying an alternative definition of cardiometabolic unhealthiness based on the presence of ≥1 metabolic abnormality; (6) applying an alternative definition of cardiometabolic unhealthiness based on the presence of ≥2 metabolic abnormalities; (7) re-including participants with prevalent cardiovascular disease at baseline; (8) additionally adjusting for alanine aminotransferase, aspartate aminotransferase, and C-reactive protein; (9) using latent class analysis to identify data-driven joint cardiometabolic–frailty phenotypes ; (10) excluding pre-frail participants to isolate the association of frailty; (11) excluding frail participants to isolate the association of pre-frailty; and (12) extending the multistate model to include death, comprising eight transitions: baseline to heart failure, baseline to atrial fibrillation, heart failure to comorbidity, atrial fibrillation to comorbidity, baseline to death, heart failure to death, atrial fibrillation to death, and comorbidity to death.

Latent class analysis identified data-driven phenotypes from five binary cardiometabolic indicators and three-level frailty status. Complete cases were randomly allocated to training (60%) and validation (40%) datasets, stratified by frailty status. One- to six-class models were fitted in the training dataset using the poLCA package. Class enumeration integrated information criteria, entropy, class-specific posterior probabilities, class prevalence, parsimony, and clinical interpretability; posterior probabilities ≥0.70 and class prevalence ≥5% served as heuristic criteria. The selected class solution was refitted in the validation dataset with the class number fixed.

Proteomic analyses were conducted among participants with available plasma proteomic data. Multivariable-adjusted plasma proteome-wide association studies (PWAS) examined associations of 2,911 proteins with cardiometabolic health and frailty status separately. Proteome-wide significance was defined using Bonferroni correction. Functional enrichment analyses of the associated proteins were performed using the Database for Annotation, Visualization and Integrated Discovery (DAVID) (https://davidbioinformatics.nih.gov/), including Gene Ontology (GO) terms—cellular component (CC), biological process (BP), and molecular function (MF)—as well as Kyoto Encyclopedia of Genes and Genomes (KEGG) and Reactome pathways. Elastic-net models with 10-fold cross-validation were then applied to PWAS-identified proteins using α values of 0.25, 0.50, 0.75 and 1.00 to assess feature-selection stability. Proteins retained across all α values were considered robustly selected. Proteins overlapping between the cardiometabolic health and frailty analyses were defined as the candidate proteomic signature of the joint phenotype.

Mediation analyses were performed using the regmedint package to assess whether candidate proteins statistically mediated the associations of CMU-F versus CMH-NF with incident AF and incident HF. The total natural indirect effect was prespecified as the primary mediation metric, and mediation proportions were reported descriptively. Within each outcome, P values were adjusted using the Benjamini–Hochberg false discovery rate procedure. Proteins with false discovery rate (FDR) <0.05 were considered statistically significant candidate mediators. To explore stage-specific molecular correlates of AF–HF progression, transition-specific mediation analyses were further conducted within the multistate framework using mstate-derived long-format data and risk sets. The robustness of proteomic findings was evaluated using protein-specific mean imputation after missingness filtering and complete-case analysis.

All statistical tests were two-sided. Bonferroni correction was applied to PWAS, and Benjamini–Hochberg false discovery rate correction was applied to multistate cohort analyses, subgroup analyses and mediation analyses, where applicable. A two-sided P value <0.05 was considered statistically significant unless otherwise specified. Analyses were performed using R (version 4.2.3).

| **Table S1. Items used to ascertain prior medication use** | | | |
| --- | --- | --- | --- |
| **Glucose-lowering agents** | | | |
| **Field ID 6153/6177 and Data-Field 20003(Data-Coding 4)** | | | |
| **Medication Name** | **Code** | **Medication Name** | **Code** |
| Insulin | 1140883066 | Orabet | 1140874690 |
| Metformin | 1140884600 | Chlorpropamide | 1140874706 |
| Glipizide | 1140874646 | Diabinese | 1140874712 |
| Glibenese | 1140874650 | Glymese | 1140874716 |
| Gliquidone | 1140874658 | Glibenclamide | 1140874718 |
| Glurenorm | 1140874660 | Daonil | 1140874724 |
| Nateglinide | 1141173882 | Acarbose | 1140868902 |
| Tolazamide | 1140874664 | Semi-daonil | 1140874726 |
| Tolanase | 1140874666 | Euglucon | 1140874728 |
| Tolbutamide | 1140874674 | Diabetamide | 1140874736 |
| Glyconon | 1140874678 | Gliclazide | 1140874744 |
| Rastinon | 1140874680 | Diamicron | 1140874746 |
| Glucophage | 1140874686 | Rosiglitazone | 1141177600 |
| Glipizide | 1140874646 | Glipizide | 1140874646 |
| Rosiglitazone + metformin | 1141189090 | Glipizide product | 1141157284 |
| Pioglitazone | 1141171646 | Troglitazone | 1141153254 |
| Glimepiride | 1141152590 |  |  |
|  |  |  |  |
| **Field ID 6153/6177** |  |  |  |
| **Antihypertensive agents** | Do you regularly take any of the following medications?  (You can select more than one answer)   - Cholesterol lowering medication - Blood pressure medication - Insulin - Hormone replacement therapy - Oral contraceptive pill or minipill - None of the above - Do not knowPrefer not to answer | | |
| **Lipid-lowering agents** |  |  |  |
|  |  |  |  |

| **Table S2. Frailty criteria** | |  | |
| --- | --- | --- | --- |
| Individual frailty phenotype components | Criteria | | UK Biobank data field |
| Weight loss | Self-reported: “Compared with one year ago, has your weight changed?”  Response: yes, lost weight = 1; other = 0;  Do not know/Prefer not to answer = missing data. | | 2306 |
| Exhaustion | Self-reported: “Over the past two weeks, how often have you felt tired or had little energy?”  Response: more than half the days or nearly every day = 1; other = 0;  Do not know/Prefer not to answer = missing data. | | 2080 |
| Slow gait speed | Self-reported: “How would you describe your usual walking pace?”  Response: slow = 1; other = 0;  Do not know/Prefer not to answer = missing data. | | 924 |
| Low grip strength | Measured grip strength expressed in kg by sex- and BMI- adjusted cut-off points. Cut-off points:  Men  If BMI ≤24.0 kg/m^2^ & grip strength ≤29 kg  If BMI 24.1 to 26.0 kg/m^2^ & grip strength ≤30 kg  If BMI 26.1 to 28.0 kg/m^2^ & grip strength ≤30 kg  If BMI >28.0 kg/m^2^ & grip strength ≤32 kg   Women  If BMI ≤23.0 kg/m^2^ & grip strength ≤17 kg  If BMI 23.1 to 26.0 kg/m^2^ & grip strength ≤17.3 kg If BMI 26.1 to 29.0 kg/m^2^ & grip strength ≤18 kg  If BMI >29.0 kg/m^2^ & grip strength ≤21 kg  If data on BMI or grip strength is not available = missing data. | | 31, 21001, 46, 47 |
| Physical activity | Self-reported: UK Biobank physical activity questionnaire. We classified the responses into: none (no physical activity in the last 4 weeks), low (light DIY activity [eg, pruning, watering the lawn] only in the past 4 weeks), medium (heavy DIY activity [eg, weeding, lawn mowing, carpentry and digging], walking for pleasure, or other exercises in the past 4 weeks), and high (strenuous sports in the past 4 weeks)  response: none or light activity with a frequency of once per week or less=1,  medium or heavy activity, or light activity more than once per week=0 | | 2624, 1011,  3637,  991, 971 |

| **Table S3. Diagnostic codes for diseases** | | | | | |
| --- | --- | --- | --- | --- | --- |
| Diseases | ICD-9 diagnosis | ICD-10 diagnosis | Self-report ^a^ | Primary Care ^b^ | Medication ^c^ / Operation code, self-reported ^d^ |
| Hypertension | 401, 4010, 4011, 4019, 402, 4020, 4021, 4029, 403, 4030, 4031, 4039, 404, 4040, 4041, 4049, 405, 4050, 4051, 4059 | I10-I13, I15 | 20002 | Category 3000 | Blood pressure medication / NA |
| Diabetes | 2500, 25000, 25001, 25009, 2501, 25011, 25019, 2503, 2504, 2505, 25099 | E10-E14 | 20002 | Category 3000 | Insulin/ NA |
| CAD | 410, 4109, 411, 4119, 412, 4129, 413, 4139, 4140, 4148, 4149 | I20-I25 | 20002 | Category 3000 | NA / Coronary angioplasty (PTCA) +/- stent; Coronary artery bypass grafts (CABG); Triple heart bypass |
| Stroke | 430-434, 436 | I60-64,Field 40001, Field 40002 | 20002 | Category 3000 | NA |
| Heart failure | 428, 4280, 4281, 4289 | I50 | 20002 | Category 3000 | NA |
| cardiomyopathy | 422, 4229, 425, 4251, 4254 | I40-I43 | 20002 | Category 3000 | NA |
| Heart valve disease | 394, 3940, 3942, 3949, 395, 3951, 3959, 396, 3969, 424, 4240, 4241, 4243, 4249 | I05-I09, I34-I38 | 20002 | Category 3000 | NA / Aortic valve repair/replacement;  Mitral valve repair/replacement;  Other valve repair/replacement |
| Arrhythmia | 426, 4260, 4261, 4263, 4264, 4265, 4266, 4267, 4269, 427, 4270, 4271, 4273, 4274, 4276, 4278, 4279 | I44, I45, I47-I49 | 20002 | Category 3000 | NA / Pacemaker; Defibrillator insertion; Cardiac ablation |

ICD: International Classification of Disease. NA, not applicable.

^a^ ICD-10 codes in the Hospital inpatient data and Death Register records

^b^ 20002 is the data code used in UK Biobank: Non-cancer illness code, self-reported.

^c^ This category contains data on primary care data recorded by health professionals working at general practices.

^d^ Data-field 6153 and 6177.

^e^ Data-Field 20004

| **Table S4. The calculation method for cumulative dietary risk** | | |
| --- | --- | --- |
| Variable | Categories reported from the touch-screen questionnaire | Binary Variables |
| Fruit & vegetables (regrouped from fruit, dried fruit & Vegetable) | Serving/day | ≥5 serving/day (Ref.)  <5 serving/day |
| Total fish intake (regrouped from Both total non-oily fish and oily fish) | Never  Less than once a week  Once a week  2-4 times a week  5-6 times a week  Once or more daily | ≥2 times a week (at least once a week of each category)  (Ref.)  < once a week of each one |
| Processed meat intake | Never  Less than once a week  Once a week  2-4 times a week  5-6 times a week  Once or more daily | ≤Once a week  (Ref.)  > Once a week |
| Red meat (regrouped from beef, pork and lamb) | Never  Less than once a week  Once a week  2-4 times a week  5-6 times a week  Once or more daily | ≤Once a week (Ref.)  >Once a week |
| Milk type used | Full cream  Semi-skimmed  Skimmed  Soya  another type of milk  Never rarely have milk | Semi-skimmed/skimmed (Ref.)  Full cream/ another type of milk/ never rarely have milk |
| Spread type | Never/rarely  Butter  Other type/ margarine  Flora pro-active/benecol | Never/rarely (Ref.)  Another selection |
| Cereal intake | Bowls/week | >5 bowls (Ref)  ≤5 bowls |
| Salt added to food | Never/rarely  Sometimes  Usually  Always | Never/rarely (Ref.)  Another selection |
| Water intake | Glasses/day | ≥6 glasses (Ref.)  <6 glasses |

“Ref.” denotes the reference (healthier) category used to calculate the cumulative dietary risk score. Each non-reference category received 1 point, yielding a total score ranging from 0 (healthiest) to 9 (least healthy).

| **Table S5. Additive and multiplicative interactions between cardiometabolic health and frailty for incident HF, incident AF, and AF–HF comorbidity progression.** | | | | | | | | | | | | |
| --- | --- | --- | --- | --- | --- | --- | --- | --- | --- | --- | --- | --- |
|  | **Multiplicative interaction(95%CI)** | **P value** | **P for FDR** | **RERI(95%CI)** | **P value** | **P for FDR** | **AP(95%CI)** | **P value** | **P for FDR** | **S(95%CI)** | **P value** | **P for FDR** |
| **Traditional Cox model** |  |  |  |  |  |  |  |  |  |  |  |  |
| HF | 1.09  (0.99, 1.20) | 0.077 |  | 0.58  (0.43, 0.73) | <0.001 |  | 0.21  (0.16, 0.26) | <0.001 |  | 1.49  (1.31, 1.69) | <0.001 |  |
| AF | 1.05  (0.99, 1.12) | 0.084 |  | 0.11  (0.04, 0.18) | 0.002 |  | 0.07  (0.03, 0.12) | 0.002 |  | 1.29  (1.07, 1.56) | 0.007 |  |
| **multistate model** |  |  |  |  |  |  |  |  |  |  |  |  |
| Baseline to HF | 1.12  (1.00, 1.25) | 0.057 | 0.230 | 0.71  (0.52, 0.90) | <0.001 | <0.001 | 0.24  (0.18, 0.30) | <0.001 | <0.001 | 1.56  (1.35, 1.81) | <0.001 | <0.001 |
| Baseline to AF | 1.04  (0.98, 1.11) | 0.188 | 0.377 | 0.09  (0.02, 0.16) | 0.015 | 0.030 | 0.06  (0.01, 0.11) | 0.014 | 0.029 | 1.25  (1.02, 1.52) | 0.028 | 0.057 |
| HF to Comorbidity | 1.10  (0.80, 1.51) | 0.545 | 0.727 | 0.12  (-0.21, 0.44) | 0.471 | 0.504 | 0.09  (-0.17, 0.36) | 0.479 | 0.504 | 1.82  (0.15,22.43) | 0.641 | 0.641 |
| AF to Comorbidit**y** | 0.98  (0.83, 1.16) | 0.795 | 0.795 | 0.08  (-0.15, 0.30) | 0.504 | 0.504 | 0.04  (-0.08, 0.17) | 0.504 | 0.504 | 1.11  (0.80, 1.54) | 0.528 | 0.641 |

Values are hazard ratios or additive-interaction measures with 95% confidence intervals. Multiplicative interaction was assessed by including a cardiometabolic health * frailty product term. Additive interaction was quantified using RERI, AP, and S, with RERI >0, AP >0, and S >1 indicating positive interaction .

Models were adjusted for age, sex, race, smoking status, drinking status, dietary score, TDI, eGFR.

Nominal P values are reported alongside P for FDR-adjusted P values, which were calculated using the Benjamini–Hochberg.

HF, heart failure; AF, atrial fibrillation; RERI, relative excess risk due to interaction; S, synergy index; TDI, Townsend deprivation index; eGFR, estimated glomerular filtration rate.

**Table S6. Cumulative transition probability matrix according to cardiometabolic health-frailty phenotype.**

| **From state** | **Predicted state** | **CMH-NF** | **CMH-F** | **CMU-NF** | **CMU-F** |
| --- | --- | --- | --- | --- | --- |
| **1-year follow up** | | | | | |
| Baseline | Baseline | 99.91% | 99.88% | 99.89% | 99.85% |
|  | HF | 0.01% | 0.01% | 0.01% | 0.02% |
|  | AF | 0.09% | 0.10% | 0.10% | 0.12% |
| HF | HF | 93.30% | 93.22% | 92.44% | 91.60% |
|  | Comorbidity | 6.70% | 6.78% | 7.56% | 8.40% |
| AF | AF | 95.42% | 93.77% | 94.04% | 92.08% |
|  | Comorbidity | 4.58% | 6.23% | 5.96% | 7.92% |
| **3-year follow up** | | | | | |
| Baseline | Baseline | 99.67% | 99.58% | 99.60% | 99.46% |
|  | HF | 0.04% | 0.06% | 0.06% | 0.10% |
|  | AF | 0.30% | 0.36% | 0.34% | 0.43% |
| HF | HF | 90.04% | 89.92% | 88.79% | 87.56% |
|  | Comorbidity | 9.96% | 10.09% | 11.21% | 12.44% |
| AF | AF | 93.33% | 90.95% | 91.34% | 88.55% |
|  | Comorbidity | 6.67% | 9.05% | 8.66% | 11.46% |
| **5-year follow up** | | | | | |
| Baseline | Baseline | 99.33% | 99.17% | 99.19% | 98.92% |
|  | HF | 0.08% | 0.12% | 0.12% | 0.23% |
|  | AF | 0.59% | 0.71% | 0.68% | 0.85% |
| HF | HF | 84.07% | 83.88% | 82.15% | 80.28% |
|  | Comorbidity | 15.93% | 16.12% | 17.85% | 19.72% |
| AF | AF | 91.27% | 88.21% | 88.71% | 85.14% |
|  | Comorbidity | 8.73% | 11.79% | 11.29% | 14.86% |
| **10-year follow up** | | | | | |
| Baseline | Baseline | 98.12% | 97.64% | 97.71% | 96.90% |
|  | HF | 0.25% | 0.41% | 0.42% | 0.76% |
|  | AF | 1.62% | 1.95% | 1.88% | 2.35% |
| HF | HF | 74.01% | 73.72% | 71.09% | 68.31% |
|  | Comorbidity | 25.99% | 26.28% | 28.91% | 31.69% |
| AF | AF | 86.33% | 81.72% | 82.46% | 77.19% |
|  | Comorbidity | 13.67% | 18.28% | 17.54% | 22.81% |
| **15-year follow up** | | | | | |
| Baseline | Baseline | 96.61% | 95.71% | 95.82% | 94.29% |
|  | HF | 0.54% | 0.88% | 0.90% | 1.61% |
|  | AF | 2.84% | 3.42% | 3.28% | 4.10% |
| HF | HF | 66.92% | 66.58% | 63.43% | 60.14% |
|  | Comorbidity | 33.08% | 33.42% | 36.57% | 39.86% |
| AF | AF | 81.21% | 75.14% | 76.10% | 69.30% |
|  | Comorbidity | 18.79% | 24.86% | 23.90% | 30.70% |

Values are model-based cumulative transition probabilities at 1, 3, 5, 10, and 15 years and are expressed as percentages. Rows indicate the starting state and predicted state within the multistate framework; HF, heart failure; AF, atrial fibrillation; CMH-NF, cardiometabolically healthy and non-frail; CMH-F, cardiometabolically healthy and pre-frail/frail; CMU-NF, cardiometabolically unhealthy and non-frail; CMU-F, cardiometabolically unhealthy and pre-frail/frail; TDI, Townsend deprivation index; eGFR, estimated glomerular filtration rate.

| **Table S7. Sensitivity Analysis I. Associations of cardiometabolic health–frailty phenotypes with incident heart failure, incident atrial fibrillation, and AF–HF disease transitions after re-including participants with cancer at baseline.** | | | | | | |
| --- | --- | --- | --- | --- | --- | --- |
|  | **N** | **Events** | **Proportion (%)** | **HR (95% CI)** | **P value** | **P for FDR** |
| **Traditional Cox model** |  |  |  |  |  |  |
| **HF** |  |  |  |  |  |  |
| CMH-F | 67179 | 1630 | 2.4 | 1.54 (1.45, 1.65) | <0.001 |  |
| CMU-NF | 86134 | 2875 | 3.3 | 1.46 (1.38, 1.55) | <0.001 |  |
| CMU-F | 72505 | 4165 | 5.7 | 2.58 (2.44, 2.72) | <0.001 |  |
| **AF** |  |  |  |  |  |  |
| CMH-F | 67179 | 3648 | 5.4 | 1.22 (1.17, 1.27) | <0.001 |  |
| CMU-NF | 86134 | 6685 | 7.8 | 1.17 (1.13, 1.21) | <0.001 |  |
| CMU-F | 72505 | 6723 | 9.3 | 1.52 (1.47, 1.58) | <0.001 |  |
| **multistate model** |  |  |  |  |  |  |
| **Baseline to HF** |  |  |  |  |  |  |
| CMH-F | 67179 | 1206 | 1.8 | 1.56 (1.44, 1.68) | <0.001 | <0.001 |
| CMU-NF | 86134 | 2072 | 2.4 | 1.48 (1.39, 1.59) | <0.001 | <0.001 |
| CMU-F | 72505 | 3110 | 4.3 | 2.69 (2.52, 2.87) | <0.001 | <0.001 |
| **Baseline to AF** |  |  |  |  |  |  |
| CMH-F | 67179 | 3280 | 4.9 | 1.20 (1.15, 1.25) | <0.001 | <0.001 |
| CMU-NF | 86134 | 5994 | 7 | 1.15 (1.11, 1.19) | <0.001 | <0.001 |
| CMU-F | 72505 | 5759 | 7.9 | 1.44 (1.39, 1.50) | <0.001 | <0.001 |
| **HF to Comorbidity** |  |  |  |  |  |  |
| CMH-F | 1206 | 368 | 30.5 | 1.02 (0.80, 1.31) | 0.868 | 0.868 |
| CMU-NF | 2072 | 691 | 33.3 | 1.17 (0.96, 1.44) | 0.127 | 0.139 |
| CMU-F | 3110 | 964 | 31 | 1.24 (1.02, 1.51) | 0.030 | 0.036 |
| **AF to Comorbidity** |  |  |  |  |  |  |
| CMH-F | 3280 | 424 | 12.9 | 1.33 (1.17, 1.51) | <0.001 | <0.001 |
| CMU-NF | 5994 | 803 | 13.4 | 1.29 (1.16, 1.44) | <0.001 | <0.001 |
| CMU-F | 5759 | 1055 | 18.3 | 1.75 (1.58, 1.95) | <0.001 | <0.001 |

The values are hazard ratios (HRs) and their 95% confidence intervals (CIs) from multivariable Cox proportional hazards models and Multistate models.

Models were adjusted for age, sex, race, smoking status, drinking status, dietary score, TDI, eGFR.

Nominal P values are reported alongside P for FDR-adjusted P values, which were calculated using the Benjamini–Hochberg.

HF, heart failure; AF, atrial fibrillation; CMH-NF, cardiometabolically healthy and non-frail; CMH-F, cardiometabolically healthy and pre-frail/frail; CMU-NF, cardiometabolically unhealthy and non-frail; CMU-F, cardiometabolically unhealthy and pre-frail/frail; TDI, Townsend deprivation index; eGFR, estimated glomerular filtration rate.

| **Table S8. Sensitivity Analysis II. Associations of cardiometabolic health–frailty phenotypes with incident heart failure, incident atrial fibrillation, and AF–HF disease transitions after excluding events occurring within the first year of follow-up.** | | | | | | |
| --- | --- | --- | --- | --- | --- | --- |
|  | **N** | **Events** | **Proportion (%)** | **HR (95% CI)** | **P value** | **P for FDR** |
| **Traditional Cox model** |  |  |  |  |  |  |
| **HF** |  |  |  |  |  |  |
| CMH-F | 60407 | 1156 | 1.9 | 1.59 (1.47, 1.72) | <0.001 |  |
| CMU-NF | 77547 | 2121 | 2.7 | 1.58 (1.48, 1.70) | <0.001 |  |
| CMU-F | 64199 | 3028 | 4.7 | 2.77 (2.60, 2.96) | <0.001 |  |
| **AF** |  |  |  |  |  |  |
| CMH-F | 60407 | 2855 | 4.7 | 1.21 (1.15, 1.27) | <0.001 |  |
| CMU-NF | 77547 | 5319 | 6.9 | 1.17 (1.13, 1.22) | <0.001 |  |
| CMU-F | 64199 | 5119 | 8 | 1.49 (1.43, 1.55) | <0.001 |  |
| **multistate model** |  |  |  |  |  |  |
| **Baseline to HF** |  |  |  |  |  |  |
| CMH-F | 60407 | 807 | 1.3 | 1.61 (1.46, 1.77) | <0.001 | <0.001 |
| CMU-NF | 77547 | 1458 | 1.9 | 1.64 (1.51, 1.79) | <0.001 | <0.001 |
| CMU-F | 64199 | 2181 | 3.4 | 2.98 (2.75, 3.22) | <0.001 | <0.001 |
| **Baseline to AF** |  |  |  |  |  |  |
| CMH-F | 60407 | 2759 | 4.6 | 1.20 (1.15, 1.26) | <0.001 | <0.001 |
| CMU-NF | 77547 | 5089 | 6.6 | 1.16 (1.12, 1.21) | <0.001 | <0.001 |
| CMU-F | 64199 | 4778 | 7.4 | 1.45 (1.39, 1.51) | <0.001 | <0.001 |
| **HF to Comorbidity** |  |  |  |  |  |  |
| CMH-F | 807 | 96 | 11.9 | 1.02 (0.78, 1.34) | 0.881 | 0.881 |
| CMU-NF | 1458 | 230 | 15.8 | 1.13 (0.91, 1.41) | 0.278 | 0.303 |
| CMU-F | 2181 | 341 | 15.6 | 1.27 (1.03, 1.57) | 0.028 | 0.034 |
| **AF to Comorbidity** |  |  |  |  |  |  |
| CMH-F | 2759 | 349 | 12.6 | 1.35 (1.17, 1.56) | <0.001 | <0.001 |
| CMU-NF | 5089 | 663 | 13 | 1.32 (1.16, 1.49) | <0.001 | <0.001 |
| CMU-F | 4778 | 847 | 17.7 | 1.78 (1.58, 2.01) | <0.001 | <0.001 |

The values are hazard ratios (HRs) and their 95% confidence intervals (CIs) from multivariable Cox proportional hazards models and Multistate models.

Models were adjusted for age, sex, race, smoking status, drinking status, dietary score, TDI, eGFR.

Nominal P values are reported alongside P for FDR-adjusted P values, which were calculated using the Benjamini–Hochberg.

HF, heart failure; AF, atrial fibrillation; CMH-NF, cardiometabolically healthy and non-frail; CMH-F, cardiometabolically healthy and pre-frail/frail; CMU-NF, cardiometabolically unhealthy and non-frail; CMU-F, cardiometabolically unhealthy and pre-frail/frail; TDI, Townsend deprivation index; eGFR, estimated glomerular filtration rate.

| **Table S9. Sensitivity Analysis III. Associations of cardiometabolic health–frailty phenotypes with incident heart failure, incident atrial fibrillation, and AF–HF disease transitions after multiple imputation of missing covariates.** | | | | | | |
| --- | --- | --- | --- | --- | --- | --- |
|  | **N** | **Events** | **Proportion (%)** | **HR (95% CI)** | **P value** | **P for FDR** |
| **Traditional Cox model** |  |  |  |  |  |  |
| **HF** |  |  |  |  |  |  |
| CMH-F | 60505 | 1180 | 2 | 1.61 (1.46-1.76) | <0.001 |  |
| CMU-NF | 77717 | 2160 | 2.8 | 1.65 (1.52-1.79) | <0.001 |  |
| CMU-F | 64390 | 3076 | 4.8 | 2.94 (2.72-3.18) | <0.001 |  |
| **AF** |  |  |  |  |  |  |
| CMH-F | 60505 | 2948 | 4.9 | 1.20 (1.15-1.26) | <0.001 |  |
| CMU-NF | 77717 | 5481 | 7.1 | 1.15 (1.11-1.20) | <0.001 |  |
| CMU-F | 64390 | 5306 | 8.2 | 1.44 (1.38-1.50) | <0.001 |  |
| **multistate model** |  |  |  |  |  |  |
| **Baseline to HF** |  |  |  |  |  |  |
| CMH-F | 60505 | 812 | 1.3 | 1.61 (1.47-1.77) | <0.001 | <0.001 |
| CMU-NF | 77717 | 1468 | 1.9 | 1.65 (1.52-1.79) | <0.001 | <0.001 |
| CMU-F | 64390 | 2187 | 3.4 | 2.98 (2.75-3.22) | <0.001 | <0.001 |
| **Baseline to AF** |  |  |  |  |  |  |
| CMH-F | 60505 | 2852 | 4.7 | 1.21 (1.15-1.26) | <0.001 | <0.001 |
| CMU-NF | 77717 | 5249 | 6.8 | 1.16 (1.11-1.20) | <0.001 | <0.001 |
| CMU-F | 64390 | 4963 | 7.7 | 1.45 (1.39-1.51) | <0.001 | <0.001 |
| **HF to Comorbidity** |  |  |  |  |  |  |
| CMH-F | 812 | 96 | 11.8 | 1.01 (0.77-1.33) | 0.930 | 0.930 |
| CMU-NF | 1468 | 232 | 15.8 | 1.13 (0.91-1.42) | 0.269 | 0.294 |
| CMU-F | 2187 | 343 | 15.7 | 1.26 (1.02-1.56) | 0.031 | 0.037 |
| **AF to Comorbidity** |  |  |  |  |  |  |
| CMH-F | 2852 | 368 | 12.9 | 1.37 (1.20-1.58) | <0.001 | <0.001 |
| CMU-NF | 5249 | 692 | 13.2 | 1.31 (1.16-1.48) | <0.001 | <0.001 |
| CMU-F | 4963 | 889 | 17.9 | 1.76 (1.57-1.98) | <0.001 | <0.001 |

The values are hazard ratios (HRs) and their 95% confidence intervals (CIs) from multivariable Cox proportional hazards models and Multistate models.

Models were adjusted for age, sex, race, smoking status, drinking status, dietary score, TDI, eGFR.

Nominal P values are reported alongside P for FDR-adjusted P values, which were calculated using the Benjamini–Hochberg.

HF, heart failure; AF, atrial fibrillation; CMH-NF, cardiometabolically healthy and non-frail; CMH-F, cardiometabolically healthy and pre-frail/frail; CMU-NF, cardiometabolically unhealthy and non-frail; CMU-F, cardiometabolically unhealthy and pre-frail/frail; TDI, Townsend deprivation index; eGFR, estimated glomerular filtration rate.

| **Table S10. Sensitivity Analysis IV. Associations of cardiometabolic health–frailty phenotypes with incident heart failure, incident atrial fibrillation, and AF–HF disease transitions among White participants** | | | | | | |
| --- | --- | --- | --- | --- | --- | --- |
|  | **N** | **Events** | **Proportion (%)** | **HR (95% CI)** | **P value** | **P for FDR** |
| **Traditional Cox model** |  |  |  |  |  |  |
| **HF** |  |  |  |  |  |  |
| CMH-F | 56477 | 1126 | 2 | 1.61 (1.48, 1.74) | <0.001 |  |
| CMU-NF | 74530 | 2097 | 2.8 | 1.58 (1.48, 1.70) | <0.001 |  |
| CMU-F | 58857 | 2866 | 4.9 | 2.76 (2.59, 2.95) | <0.001 |  |
| **AF** |  |  |  |  |  |  |
| CMH-F | 56477 | 2871 | 5.1 | 1.22 (1.16, 1.27) | <0.001 |  |
| CMU-NF | 74530 | 5374 | 7.2 | 1.17 (1.12, 1.22) | <0.001 |  |
| CMU-F | 58857 | 5093 | 8.7 | 1.50 (1.44, 1.56) | <0.001 |  |
| **multistate model** |  |  |  |  |  |  |
| **Baseline to HF** |  |  |  |  |  |  |
| CMH-F | 56477 | 767 | 1.4 | 1.62 (1.47, 1.78) | <0.001 | <0.001 |
| CMU-NF | 74530 | 1422 | 1.9 | 1.66 (1.52, 1.80) | <0.001 | <0.001 |
| CMU-F | 58857 | 2016 | 3.4 | 2.98 (2.75, 3.23) | <0.001 | <0.001 |
| **Baseline to AF** |  |  |  |  |  |  |
| CMH-F | 56477 | 2782 | 4.9 | 1.21 (1.16, 1.27) | <0.001 | <0.001 |
| CMU-NF | 74530 | 5149 | 6.9 | 1.16 (1.11, 1.20) | <0.001 | <0.001 |
| CMU-F | 58857 | 4774 | 8.1 | 1.46 (1.40, 1.52) | <0.001 | <0.001 |
| **HF to Comorbidity** |  |  |  |  |  |  |
| CMH-F | 767 | 89 | 11.6 | 0.97 (0.74, 1.28) | 0.835 | 0.835 |
| CMU-NF | 1422 | 225 | 15.8 | 1.10 (0.88, 1.38) | 0.386 | 0.422 |
| CMU-F | 2016 | 319 | 15.8 | 1.24 (1.00, 1.54) | 0.046 | 0.055 |
| **AF to Comorbidity** |  |  |  |  |  |  |
| CMH-F | 2782 | 359 | 12.9 | 1.37 (1.19, 1.58) | <0.001 | <0.001 |
| CMU-NF | 5149 | 675 | 13.1 | 1.31 (1.16, 1.48) | <0.001 | <0.001 |
| CMU-F | 4774 | 850 | 17.8 | 1.76 (1.56, 1.97) | <0.001 | <0.001 |

The values are hazard ratios (HRs) and their 95% confidence intervals (CIs) from multivariable Cox proportional hazards models and Multistate models.

Models were adjusted for age, sex, race, smoking status, drinking status, dietary score, TDI, eGFR.

Nominal P values are reported alongside P for FDR-adjusted P values, which were calculated using the Benjamini–Hochberg.

HF, heart failure; AF, atrial fibrillation; CMH-NF, cardiometabolically healthy and non-frail; CMH-F, cardiometabolically healthy and pre-frail/frail; CMU-NF, cardiometabolically unhealthy and non-frail; CMU-F, cardiometabolically unhealthy and pre-frail/frail; TDI, Townsend deprivation index; eGFR, estimated glomerular filtration rate.

| **Table S11. Sensitivity Analysis Ⅴ.Associations of cardiometabolic health–frailty phenotypes with incident heart failure, incident atrial fibrillation, and AF–HF disease transitions using ≥1 metabolic abnormality to define cardiometabolic unhealthiness.** | | | | | | |
| --- | --- | --- | --- | --- | --- | --- |
|  | **N** | **Events** | **Proportion (%)** | **HR (95% CI)** | **P value** | **P for FDR** |
| **Traditional Cox model** |  |  |  |  |  |  |
| **HF** |  |  |  |  |  |  |
| CMH-F | 8551 | 66 | 0.8 | 1.62 (1.18, 2.22) | 0.003 |  |
| CMU-NF | 172918 | 3463 | 2 | 2.41 (1.96, 2.96) | <0.001 |  |
| CMU-F | 116344 | 4190 | 3.6 | 4.23 (3.45, 5.20) | <0.001 |  |
| **AF** |  |  |  |  |  |  |
| CMH-F | 8551 | 229 | 2.7 | 1.18 (1.01, 1.38) | 0.040 |  |
| CMU-NF | 172918 | 9903 | 5.7 | 1.40 (1.27, 1.53) | <0.001 |  |
| CMU-F | 116344 | 8025 | 6.9 | 1.77 (1.61, 1.94) | <0.001 |  |
| **multistate model** |  |  |  |  |  |  |
| **Baseline to HF** |  |  |  |  |  |  |
| CMH-F | 8551 | 53 | 0.6 | 1.70 (1.19, 2.42) | 0.004 | 0.006 |
| CMU-NF | 172918 | 2340 | 1.4 | 2.30 (1.82, 2.92) | <0.001 | <0.001 |
| CMU-F | 116344 | 2946 | 2.5 | 4.17 (3.29, 5.29) | <0.001 | <0.001 |
| **Baseline to AF** |  |  |  |  |  |  |
| CMH-F | 8551 | 220 | 2.6 | 1.15 (0.98, 1.35) | 0.085 | 0.127 |
| CMU-NF | 172918 | 9557 | 5.5 | 1.38 (1.26, 1.51) | <0.001 | <0.001 |
| CMU-F | 116344 | 7595 | 6.5 | 1.72 (1.57, 1.89) | <0.001 | <0.001 |
| **HF to Comorbidity** |  |  |  |  |  |  |
| CMH-F | 53 | 9 | 17 | 2.26 (0.80, 6.39) | 0.125 | 0.166 |
| CMU-NF | 2340 | 346 | 14.8 | 1.21 (0.54, 2.71) | 0.648 | 0.648 |
| CMU-F | 2946 | 430 | 14.6 | 1.32 (0.59, 2.97) | 0.500 | 0.600 |
| **AF to Comorbidity** |  |  |  |  |  |  |
| CMH-F | 220 | 13 | 5.9 | 1.22 (0.62, 2.41) | 0.567 | 0.618 |
| CMU-NF | 9557 | 1123 | 11.8 | 2.05 (1.36, 3.10) | <0.001 | 0.001 |
| CMU-F | 7595 | 1244 | 16.4 | 2.83 (1.87, 4.28) | <0.001 | <0.001 |

The values are hazard ratios (HRs) and their 95% confidence intervals (CIs) from multivariable Cox proportional hazards models and Multistate models.

Models were adjusted for age, sex, race, smoking status, drinking status, dietary score, TDI, eGFR.

Nominal P values are reported alongside P for FDR-adjusted P values, which were calculated using the Benjamini–Hochberg.

HF, heart failure; AF, atrial fibrillation; CMH-NF, cardiometabolically healthy and non-frail; CMH-F, cardiometabolically healthy and pre-frail/frail; CMU-NF, cardiometabolically unhealthy and non-frail; CMU-F, cardiometabolically unhealthy and pre-frail/frail; TDI, Townsend deprivation index; eGFR, estimated glomerular filtration rate.

| **Table S12. Sensitivity Analysis Ⅵ. Associations of cardiometabolic health–frailty phenotypes with incident heart failure, incident atrial fibrillation, and AF–HF disease transitions using ≥2 metabolic abnormalities to define cardiometabolic unhealthiness.** | | | | | | |
| --- | --- | --- | --- | --- | --- | --- |
|  | **N** | **Events** | **Proportion (%)** | **HR (95% CI)** | **P value** | **P for FDR** |
| **Traditional Cox model** |  |  |  |  |  |  |
| **HF** |  |  |  |  |  |  |
| CMH-F | 29185 | 364 | 1.2 | 1.45 (1.27, 1.66) | <0.001 |  |
| CMU-NF | 130367 | 3018 | 2.3 | 1.76 (1.60, 1.93) | <0.001 |  |
| CMU-F | 95710 | 3892 | 4.1 | 3.07 (2.81, 3.37) | <0.001 |  |
| **AF** |  |  |  |  |  |  |
| CMH-F | 29185 | 1064 | 3.6 | 1.14 (1.06, 1.23) | <0.001 |  |
| CMU-NF | 130367 | 8258 | 6.3 | 1.25 (1.19, 1.31) | <0.001 |  |
| CMU-F | 95710 | 7190 | 7.5 | 1.56 (1.49, 1.64) | <0.001 |  |
| **multistate model** |  |  |  |  |  |  |
| **Baseline to HF** |  |  |  |  |  |  |
| CMH-F | 29185 | 261 | 0.9 | 1.48 (1.27, 1.74) | <0.001 | <0.001 |
| CMU-NF | 130367 | 2040 | 1.6 | 1.81 (1.62, 2.02) | <0.001 | <0.001 |
| CMU-F | 95710 | 2738 | 2.9 | 3.26 (2.92, 3.64) | <0.001 | <0.001 |
| **Baseline to AF** |  |  |  |  |  |  |
| CMH-F | 29185 | 1036 | 3.5 | 1.14 (1.05, 1.22) | <0.001 | 0.001 |
| CMU-NF | 130367 | 7947 | 6.1 | 1.23 (1.17, 1.29) | <0.001 | <0.001 |
| CMU-F | 95710 | 6779 | 7.1 | 1.52 (1.45, 1.60) | <0.001 | <0.001 |
| **HF to Comorbidity** |  |  |  |  |  |  |
| CMH-F | 261 | 28 | 10.7 | 1.07 (0.66, 1.74) | 0.776 | 0.776 |
| CMU-NF | 2040 | 311 | 15.2 | 1.19 (0.86, 1.66) | 0.292 | 0.318 |
| CMU-F | 2738 | 411 | 15 | 1.31 (0.95, 1.81) | 0.102 | 0.123 |
| **AF to Comorbidity** |  |  |  |  |  |  |
| CMH-F | 1036 | 103 | 9.9 | 1.29 (1.01, 1.65) | 0.040 | 0.054 |
| CMU-NF | 7947 | 978 | 12.3 | 1.38 (1.17, 1.63) | <0.001 | <0.001 |
| CMU-F | 6779 | 1154 | 17 | 1.90 (1.61, 2.24) | <0.001 | <0.001 |

The values are hazard ratios (HRs) and their 95% confidence intervals (CIs) from multivariable Cox proportional hazards models and Multistate models.

Models were adjusted for age, sex, race, smoking status, drinking status, dietary score, TDI, eGFR.

Nominal P values are reported alongside P for FDR-adjusted P values, which were calculated using the Benjamini–Hochberg.

HF, heart failure; AF, atrial fibrillation; CMH-NF, cardiometabolically healthy and non-frail; CMH-F, cardiometabolically healthy and pre-frail/frail; CMU-NF, cardiometabolically unhealthy and non-frail; CMU-F, cardiometabolically unhealthy and pre-frail/frail; TDI, Townsend deprivation index; eGFR, estimated glomerular filtration rate.

| **Table S13. Sensitivity Analysis Ⅶ. Associations of cardiometabolic health–frailty phenotypes with incident heart failure, incident atrial fibrillation, and AF–HF disease transitions after re-including participants with prevalent cardiovascular disease at baseline.** | | | | | | |
| --- | --- | --- | --- | --- | --- | --- |
|  | **N** | **Events** | **Proportion (%)** | **HR (95% CI)** | **P value** | **P for FDR** |
| **Traditional Cox model** |  |  |  |  |  |  |
| **HF** |  |  |  |  |  |  |
| CMH-F | 62743 | 1348 | 2.1 | 1.65 (1.54, 1.78) | <0.001 |  |
| CMU-NF | 87054 | 3140 | 3.6 | 1.83 (1.72, 1.95) | <0.001 |  |
| CMU-F | 76569 | 5046 | 6.6 | 3.37 (3.18, 3.57) | <0.001 |  |
| **AF** |  |  |  |  |  |  |
| CMH-F | 62743 | 3274 | 5.2 | 1.21 (1.16, 1.27) | <0.001 |  |
| CMU-NF | 87054 | 7078 | 8.1 | 1.23 (1.19, 1.27) | <0.001 |  |
| CMU-F | 76569 | 7650 | 10 | 1.61 (1.55, 1.67) | <0.001 |  |
| **multistate model** |  |  |  |  |  |  |
| **Baseline to HF** |  |  |  |  |  |  |
| CMH-F | 62743 | 918 | 1.5 | 1.67 (1.53, 1.83) | <0.001 | <0.001 |
| CMU-NF | 87054 | 2159 | 2.5 | 1.96 (1.82, 2.12) | <0.001 | <0.001 |
| CMU-F | 76569 | 3580 | 4.7 | 3.71 (3.45, 3.98) | <0.001 | <0.001 |
| **Baseline to AF** |  |  |  |  |  |  |
| CMH-F | 62743 | 3157 | 5 | 1.21 (1.15, 1.26) | <0.001 | <0.001 |
| CMU-NF | 87054 | 6710 | 7.7 | 1.21 (1.17, 1.26) | <0.001 | <0.001 |
| CMU-F | 76569 | 6987 | 9.1 | 1.54 (1.49, 1.60) | <0.001 | <0.001 |
| **HF to Comorbidity** |  |  |  |  |  |  |
| CMH-F | 918 | 117 | 12.7 | 1.02 (0.79, 1.30) | 0.906 | 0.906 |
| CMU-NF | 2159 | 368 | 17 | 1.06 (0.87, 1.30) | 0.560 | 0.611 |
| CMU-F | 3580 | 663 | 18.5 | 1.20 (0.99, 1.45) | 0.058 | 0.069 |
| **AF to Comorbidity** |  |  |  |  |  |  |
| CMH-F | 3157 | 430 | 13.6 | 1.41 (1.23, 1.60) | <0.001 | <0.001 |
| CMU-NF | 6710 | 981 | 14.6 | 1.36 (1.22, 1.52) | <0.001 | <0.001 |
| CMU-F | 6987 | 1466 | 21 | 1.94 (1.75, 2.16) | <0.001 | <0.001 |

The values are hazard ratios (HRs) and their 95% confidence intervals (CIs) from multivariable Cox proportional hazards models and Multistate models.

Models were adjusted for age, sex, race, smoking status, drinking status, dietary score, TDI, eGFR.

Nominal P values are reported alongside P for FDR-adjusted P values, which were calculated using the Benjamini–Hochberg.

HF, heart failure; AF, atrial fibrillation; CMH-NF, cardiometabolically healthy and non-frail; CMH-F, cardiometabolically healthy and pre-frail/frail; CMU-NF, cardiometabolically unhealthy and non-frail; CMU-F, cardiometabolically unhealthy and pre-frail/frail; TDI, Townsend deprivation index; eGFR, estimated glomerular filtration rate.

| **Table S14. Sensitivity Analysis Ⅷ. Associations of cardiometabolic health–frailty phenotypes with incident heart failure, incident atrial fibrillation, and AF–HF disease transitions after additional adjustment for alanine aminotransferase, aspartate aminotransferase, and C-reactive protein.** | | | | | | |
| --- | --- | --- | --- | --- | --- | --- |
|  | **N** | **Events** | **Proportion (%)** | **HR (95% CI)** | **P value** | **P for FDR** |
| **Traditional Cox model** |  |  |  |  |  |  |
| **HF** |  |  |  |  |  |  |
| CMH-F | 60505 | 1180 | 2 | 1.57 (1.45, 1.70) | <0.001 |  |
| CMU-NF | 77717 | 2160 | 2.8 | 1.55 (1.45, 1.66) | <0.001 |  |
| CMU-F | 64390 | 3076 | 4.8 | 2.64 (2.47, 2.81) | <0.001 |  |
| **AF** |  |  |  |  |  |  |
| CMH-F | 60505 | 2948 | 4.9 | 1.19 (1.14, 1.25) | <0.001 |  |
| CMU-NF | 77717 | 5481 | 7.1 | 1.16 (1.12, 1.21) | <0.001 |  |
| CMU-F | 64390 | 5306 | 8.2 | 1.44 (1.39, 1.50) | <0.001 |  |
| **multistate model** |  |  |  |  |  |  |
| **Baseline to HF** |  |  |  |  |  |  |
| CMH-F | 60505 | 812 | 1.3 | 1.58 (1.44, 1.74) | <0.001 | <0.001 |
| CMU-NF | 77717 | 1468 | 1.9 | 1.61 (1.48, 1.75) | <0.001 | <0.001 |
| CMU-F | 64390 | 2187 | 3.4 | 2.84 (2.63, 3.07) | <0.001 | <0.001 |
| **Baseline to AF** |  |  |  |  |  |  |
| CMH-F | 60505 | 2852 | 4.7 | 1.18 (1.13, 1.24) | <0.001 | <0.001 |
| CMU-NF | 77717 | 5249 | 6.8 | 1.15 (1.10, 1.20) | <0.001 | <0.001 |
| CMU-F | 64390 | 4963 | 7.7 | 1.41 (1.35, 1.46) | <0.001 | <0.001 |
| **HF to Comorbidity** |  |  |  |  |  |  |
| CMH-F | 812 | 96 | 11.8 | 1.01 (0.77, 1.32) | 0.939 | 0.939 |
| CMU-NF | 1468 | 232 | 15.8 | 1.14 (0.91, 1.42) | 0.263 | 0.287 |
| CMU-F | 2187 | 343 | 15.7 | 1.26 (1.02, 1.57) | 0.035 | 0.042 |
| **AF to Comorbidity** |  |  |  |  |  |  |
| CMH-F | 2852 | 368 | 12.9 | 1.35 (1.17, 1.55) | <0.001 | <0.001 |
| CMU-NF | 5249 | 692 | 13.2 | 1.30 (1.15, 1.47) | <0.001 | <0.001 |
| CMU-F | 4963 | 889 | 17.9 | 1.71 (1.52, 1.92) | <0.001 | <0.001 |

The values are hazard ratios (HRs) and their 95% confidence intervals (CIs) from multivariable Cox proportional hazards models and Multistate models.

Models were adjusted for age, sex, race, smoking status, drinking status, dietary score, TDI, eGFR.

Nominal P values are reported alongside P for FDR-adjusted P values, which were calculated using the Benjamini–Hochberg.

HF, heart failure; AF, atrial fibrillation; CMH-NF, cardiometabolically healthy and non-frail; CMH-F, cardiometabolically healthy and pre-frail/frail; CMU-NF, cardiometabolically unhealthy and non-frail; CMU-F, cardiometabolically unhealthy and pre-frail/frail; TDI, Townsend deprivation index; eGFR, estimated glomerular filtration rate.

| **Table S15. Sensitivity Analysis Ⅸ. Associations of latent class–derived cardiometabolic–frailty phenotypes with incident heart failure, incident atrial fibrillation, and AF–HF disease transitions.** | | | | | | |
| --- | --- | --- | --- | --- | --- | --- |
|  | **N** | **Events** | **Proportion (%)** | **HR (95% CI)** | **P value** | **P for FDR** |
| **Training** |  |  |  |  |  |  |
| **Traditional Cox model** |  |  |  |  |  |  |
| **HF** |  |  |  |  |  |  |
| Class 2 | 84629 | 2119 | 2.50 | 1.75 (1.60, 1.91) | <0.001 |  |
| Class 3 | 45229 | 1919 | 4.24 | 2.38 (2.18, 2.61) | <0.001 |  |
| **AF** |  |  |  |  |  |  |
| Class 2 | 84629 | 5240 | 6.19 | 1.32 (1.25, 1.39) | <0.001 |  |
| Class 3 | 45229 | 3691 | 8.16 | 1.44 (1.36, 1.52) | <0.001 |  |
| **multistate model** |  |  |  |  |  |  |
| **Baseline to HF** |  |  |  |  |  |  |
| Class 2 | 84480 | 1314 | 1.56 | 1.80 (1.61, 2.01) | <0.001 | <0.001 |
| Class 3 | 45081 | 1222 | 2.71 | 2.57 (2.29, 2.88) | <0.001 | <0.001 |
| **Baseline to AF** |  |  |  |  |  |  |
| Class 2 | 84480 | 5005 | 5.92 | 1.30 (1.24, 1.37) | <0.001 | <0.001 |
| Class 3 | 45081 | 3462 | 7.68 | 1.41 (1.33, 1.49) | <0.001 | <0.001 |
| **HF to Comorbidity** |  |  |  |  |  |  |
| Class 2 | 1314 | 207 | 15.75 | 1.32 (0.97, 1.81) | 0.081 | 0.081 |
| Class 3 | 1222 | 216 | 17.68 | 1.45 (1.06, 1.99) | 0.019 | 0.022 |
| **AF to Comorbidity** |  |  |  |  |  |  |
| Class 2 | 5005 | 684 | 13.67 | 1.35 (1.15, 1.58) | <0.001 | <0.001 |
| Class 3 | 3462 | 562 | 16.23 | 1.53 (1.29, 1.80) | <0.001 | <0.001 |
| **Validation** |  |  |  |  |  |  |
| **Traditional Cox model** |  |  |  |  |  |  |
| **HF** |  |  |  |  |  |  |
| Class 2 | 59909 | 1406 | 2.35 | 1.71 (1.53, 1.92) | <0.001 |  |
| Class 3 | 30690 | 1319 | 4.30 | 2.44 (2.17, 2.73) | <0.001 |  |
| **AF** |  |  |  |  |  |  |
| Class 2 | 59909 | 3547 | 5.92 | 1.31 (1.23, 1.39) | <0.001 |  |
| Class 3 | 30690 | 2635 | 8.59 | 1.54 (1.44, 1.64) | <0.001 |  |
| **multistate model** |  |  |  |  |  |  |
| **Baseline to HF** |  |  |  |  |  |  |
| Class 2 | 59809 | 883 | 1.48 | 1.73 (1.51, 2.00) | <0.001 | <0.001 |
| Class 3 | 30617 | 851 | 2.78 | 2.61 (2.26, 3.01) | <0.001 | <0.001 |
| **Baseline to AF** |  |  |  |  |  |  |
| Class 2 | 59809 | 3381 | 5.65 | 1.29 (1.21, 1.38) | <0.001 | <0.001 |
| Class 3 | 30617 | 2480 | 8.10 | 1.51 (1.41, 1.62) | <0.001 | <0.001 |
| **HF to Comorbidity** |  |  |  |  |  |  |
| Class 2 | 883 | 145 | 16.42 | 1.39 (0.93, 2.08) | 0.113 | 0.113 |
| Class 3 | 851 | 145 | 17.04 | 1.44 (0.95, 2.17) | 0.084 | 0.096 |
| **AF to Comorbidity** |  |  |  |  |  |  |
| Class 2 | 3381 | 444 | 13.13 | 1.48 (1.20, 1.82) | <0.001 | <0.001 |
| Class 3 | 2480 | 405 | 16.33 | 1.72 (1.39, 2.13) | <0.001 | <0.001 |

The values are hazard ratios (HRs) and their 95% confidence intervals (CIs) from multivariable Cox proportional hazards models and Multistate models. Latent classes were identified from five binary cardiometabolic indicators—adiposity, dysglycaemia, elevated blood pressure, hypertriglyceridaemia, and low high-density lipoprotein cholesterol—and three-level frailty status (robust, pre-frail, or frail). Class 1 was characterised by the lowest overall cardiometabolic and frailty burden; Class 2 by predominant adiposity and elevated blood pressure, with intermediate hypertriglyceridaemia and pre-frailty; and Class 3 by concurrent enrichment of multiple cardiometabolic abnormalities and frailty. Class 1 served as the reference group in both the training and validation datasets. Models were adjusted for age, sex, race, smoking status, drinking status, dietary score, TDI, eGFR.Nominal P values are reported alongside P for FDR-adjusted P values, which were calculated using the Benjamini–Hochberg.HF, heart failure; AF, atrial fibrillation; TDI, Townsend deprivation index; eGFR, estimated glomerular filtration rate.

| **Table S16. Sensitivity Analysis Ⅹ. Associations of cardiometabolic health–frailty phenotypes with incident heart failure, incident atrial fibrillation, and AF–HF disease transitions after excluding pre-frail participants.** | | | | | | |
| --- | --- | --- | --- | --- | --- | --- |
|  | **N** | **Events** | **Proportion (%)** | **HR (95% CI)** | **P value** | **P for FDR** |
| **Traditional Cox model** |  |  |  |  |  |  |
| **HF** |  |  |  |  |  |  |
| CMH-F | 2907 | 132 | 4.5 | 3.57 (2.98, 4.28) | <0.001 |  |
| CMU-NF | 77717 | 2160 | 2.8 | 1.57 (1.47, 1.69) | <0.001 |  |
| CMU-F | 5842 | 533 | 9.1 | 5.52 (4.96, 6.14) | <0.001 |  |
| **AF** |  |  |  |  |  |  |
| CMH-F | 2907 | 218 | 7.5 | 2.02 (1.77, 2.32) | <0.001 |  |
| CMU-NF | 77717 | 5481 | 7.1 | 1.16 (1.11, 1.21) | <0.001 |  |
| CMU-F | 5842 | 634 | 10.9 | 2.24 (2.06, 2.44) | <0.001 |  |
| **multistate model** |  |  |  |  |  |  |
| **Baseline to HF** |  |  |  |  |  |  |
| CMH-F | 2907 | 99 | 3.4 | 3.91 (3.17, 4.83) | <0.001 | <0.001 |
| CMU-NF | 77717 | 1468 | 1.9 | 1.64 (1.51, 1.78) | <0.001 | <0.001 |
| CMU-F | 5842 | 398 | 6.8 | 6.26 (5.52, 7.10) | <0.001 | <0.001 |
| **Baseline to AF** |  |  |  |  |  |  |
| CMH-F | 2907 | 204 | 7 | 1.97 (1.71, 2.27) | <0.001 | <0.001 |
| CMU-NF | 77717 | 5249 | 6.8 | 1.15 (1.10, 1.19) | <0.001 | <0.001 |
| CMU-F | 5842 | 564 | 9.7 | 2.11 (1.93, 2.31) | <0.001 | <0.001 |
| **HF to Comorbidity** |  |  |  |  |  |  |
| CMH-F | 99 | 14 | 14.1 | 1.50 (0.86, 2.64) | 0.157 | 0.171 |
| CMU-NF | 1468 | 232 | 15.8 | 1.14 (0.92, 1.43) | 0.236 | 0.236 |
| CMU-F | 398 | 70 | 17.6 | 1.49 (1.09, 2.04) | 0.013 | 0.016 |
| **AF to Comorbidity** |  |  |  |  |  |  |
| CMH-F | 204 | 33 | 16.2 | 1.58 (1.10, 2.27) | 0.012 | 0.016 |
| CMU-NF | 5249 | 692 | 13.2 | 1.32 (1.17, 1.49) | <0.001 | <0.001 |
| CMU-F | 564 | 135 | 23.9 | 2.33 (1.89, 2.86) | <0.001 | <0.001 |

The values are hazard ratios (HRs) and their 95% confidence intervals (CIs) from multivariable Cox proportional hazards models and Multistate models.

Models were adjusted for age, sex, race, smoking status, drinking status, dietary score, TDI, eGFR.

Nominal P values are reported alongside P for FDR-adjusted P values, which were calculated using the Benjamini–Hochberg.

HF, heart failure; AF, atrial fibrillation; CMH-NF, cardiometabolically healthy and non-frail; CMH-F, cardiometabolically healthy and frail; CMU-NF, cardiometabolically unhealthy and non-frail; CMU-F, cardiometabolically unhealthy and frail; TDI, Townsend deprivation index; eGFR, estimated glomerular filtration rate.

| **Table S17. Sensitivity Analysis ⅩⅠ. Associations of cardiometabolic health–frailty phenotypes with incident heart failure, incident atrial fibrillation, and AF–HF disease transitions after excluding frail participants.** | | | | | | |
| --- | --- | --- | --- | --- | --- | --- |
|  | **N** | **Events** | **Proportion (%)** | **HR (95% CI)** | **P value** | **P for FDR** |
| **Traditional Cox model** |  |  |  |  |  |  |
| **HF** |  |  |  |  |  |  |
| CMH-F | 57598 | 1048 | 1.8 | 1.51 (1.40, 1.64) | <0.001 |  |
| CMU-NF | 77717 | 2160 | 2.8 | 1.57 (1.47, 1.68) | <0.001 |  |
| CMU-F | 58548 | 2543 | 4.3 | 2.52 (2.36, 2.69) | <0.001 |  |
| **AF** |  |  |  |  |  |  |
| CMH-F | 57598 | 2730 | 4.7 | 1.18 (1.12, 1.23) | <0.001 |  |
| CMU-NF | 77717 | 5481 | 7.1 | 1.17 (1.13, 1.22) | <0.001 |  |
| CMU-F | 58548 | 4672 | 8 | 1.44 (1.38, 1.50) | <0.001 |  |
| **multistate model** |  |  |  |  |  |  |
| **Baseline to HF** |  |  |  |  |  |  |
| CMH-F | 57598 | 713 | 1.2 | 1.51 (1.37, 1.66) | <0.001 | <0.001 |
| CMU-NF | 77717 | 1468 | 1.9 | 1.63 (1.50, 1.78) | <0.001 | <0.001 |
| CMU-F | 58548 | 1789 | 3.1 | 2.69 (2.48, 2.91) | <0.001 | <0.001 |
| **Baseline to AF** |  |  |  |  |  |  |
| CMH-F | 57598 | 2648 | 4.6 | 1.17 (1.12, 1.23) | <0.001 | <0.001 |
| CMU-NF | 77717 | 5249 | 6.8 | 1.16 (1.11, 1.20) | <0.001 | <0.001 |
| CMU-F | 58548 | 4399 | 7.5 | 1.40 (1.35, 1.46) | <0.001 | <0.001 |
| **HF to Comorbidity** |  |  |  |  |  |  |
| CMH-F | 713 | 82 | 11.5 | 0.96 (0.73, 1.28) | 0.799 | 0.799 |
| CMU-NF | 1468 | 232 | 15.8 | 1.14 (0.91, 1.42) | 0.258 | 0.281 |
| CMU-F | 1789 | 273 | 15.3 | 1.25 (1.00, 1.55) | 0.047 | 0.056 |
| **AF to Comorbidity** |  |  |  |  |  |  |
| CMH-F | 2648 | 335 | 12.7 | 1.36 (1.18, 1.57) | <0.001 | <0.001 |
| CMU-NF | 5249 | 692 | 13.2 | 1.31 (1.16, 1.48) | <0.001 | <0.001 |
| CMU-F | 4399 | 754 | 17.1 | 1.69 (1.51, 1.91) | <0.001 | <0.001 |

The values are hazard ratios (HRs) and their 95% confidence intervals (CIs) from multivariable Cox proportional hazards models and Multistate models.

Models were adjusted for age, sex, race, smoking status, drinking status, dietary score, TDI, eGFR.

Nominal P values are reported alongside P for FDR-adjusted P values, which were calculated using the Benjamini–Hochberg.

HF, heart failure; AF, atrial fibrillation; CMH-NF, cardiometabolically healthy and non-frail; CMH-F, cardiometabolically healthy and pre-frail; CMU-NF, cardiometabolically unhealthy and non-frail; CMU-F, cardiometabolically unhealthy and pre-frail; TDI, Townsend deprivation index; eGFR, estimated glomerular filtration rate.

| **Table S18. Sensitivity Analysis ⅩⅡ. Associations of cardiometabolic health–frailty phenotypes with cardiovascular disease and mortality transitions in the death-inclusive multistate model.** | | | | | | |
| --- | --- | --- | --- | --- | --- | --- |
|  | **N** | **Events** | **Proportion (%)** | **HR (95% CI)** | **P value** | **P for FDR** |
| **Baseline to HF** |  |  |  |  |  |  |
| CMH-F | 60505 | 812 | 1.3 | 1.61 (1.47, 1.77) | <0.001 | <0.001 |
| CMU-NF | 77717 | 1468 | 1.9 | 1.65 (1.52, 1.79) | <0.001 | <0.001 |
| CMU-F | 64390 | 2187 | 3.4 | 2.98 (2.76, 3.22) | <0.001 | <0.001 |
| **Baseline to AF** |  |  |  |  |  |  |
| CMH-F | 60505 | 2852 | 4.7 | 1.20 (1.15, 1.26) | <0.001 | <0.001 |
| CMU-NF | 77717 | 5249 | 6.8 | 1.16 (1.11, 1.20) | <0.001 | <0.001 |
| CMU-F | 64390 | 4963 | 7.7 | 1.45 (1.39, 1.51) | <0.001 | <0.001 |
| **HF to Comorbidity** |  |  |  |  |  |  |
| CMH-F | 812 | 96 | 11.8 | 1.01 (0.77, 1.33) | 0.925 | 0.925 |
| CMU-NF | 1468 | 232 | 15.8 | 1.13 (0.91, 1.42) | 0.269 | 0.294 |
| CMU-F | 2187 | 343 | 15.7 | 1.27 (1.02, 1.57) | 0.030 | 0.035 |
| **AF to Comorbidity** |  |  |  |  |  |  |
| CMH-F | 2852 | 368 | 12.9 | 1.37 (1.20, 1.58) | <0.001 | <0.001 |
| CMU-NF | 5249 | 692 | 13.2 | 1.31 (1.16, 1.48) | <0.001 | <0.001 |
| CMU-F | 4963 | 889 | 17.9 | 1.76 (1.57, 1.98) | <0.001 | <0.001 |
| **Baseline to death** |  |  |  |  |  |  |
| CMH-F | 60505 | 3823 | 6.3 | 1.38 (1.32, 1.44) | <0.001 | <0.001 |
| CMU-NF | 77717 | 5791 | 7.5 | 1.19 (1.15, 1.24) | <0.001 | <0.001 |
| CMU-F | 64390 | 6272 | 9.7 | 1.60 (1.54, 1.66) | <0.001 | <0.001 |
| **HF to death** |  |  |  |  |  |  |
| CMH-F | 812 | 316 | 38.9 | 1.28 (1.09, 1.50) | 0.002 | 0.003 |
| CMU-NF | 1468 | 488 | 33.2 | 0.97 (0.84, 1.12) | 0.091 | 0.726 |
| CMU-F | 2187 | 858 | 39.2 | 1.23 (1.08, 1.41) | 0.002 | 0.003 |
| **AF to death** |  |  |  |  |  |  |
| CMH-F | 2852 | 489 | 17.1 | 1.26 (1.12, 1.42) | <0.001 | <0.001 |
| CMU-NF | 5249 | 942 | 17.9 | 1.18 (1.07, 1.31) | 0.001 | 0.002 |
| CMU-F | 4963 | 1046 | 21.1 | 1.38 (1.25, 1.52) | <0.001 | <0.001 |
| **Comorbidity to death** |  |  |  |  |  |  |
| CMH-F | 464 | 212 | 45.7 | 1.43 (1.18, 1.74) | <0.001 | <0.001 |
| CMU-NF | 924 | 376 | 40.7 | 1.12 (0.94, 1.33) | 0.201 | 0.230 |
| CMU-F | 1232 | 624 | 50.6 | 1.41 (1.20, 1.66) | <0.001 | <0.001 |

The values are hazard ratios (HRs) and their 95% confidence intervals (CIs) from multivariable Cox proportional hazards models and Multistate models.

Models were adjusted for age, sex, race, smoking status, drinking status, dietary score, TDI, eGFR.

Nominal P values are reported alongside P for FDR-adjusted P values, which were calculated using the Benjamini–Hochberg.

HF, heart failure; AF, atrial fibrillation; CMH-NF, cardiometabolically healthy and non-frail; CMH-F, cardiometabolically healthy and pre-frail/frail; CMU-NF, cardiometabolically unhealthy and non-frail; CMU-F, cardiometabolically unhealthy and pre-frail/frail; TDI, Townsend deprivation index; eGFR, estimated glomerular filtration rate.

| **Table S19. Baseline Characteristics of the Proteomics Subcohort** | | | | | |
| --- | --- | --- | --- | --- | --- |
| **Variable** | **Overall (N=28,206)** | **CMH-NF (N=10,071)** | **CMH-F (N=5,405)** | **CMU-NF (N=6,872)** | **CMU-F (N=5,858)** |
| **Age** | 56.04 (8.19) | 54.52 (8.14) | 54.55 (8.19) | 57.88 (7.82) | 57.85 (7.86) |
| **Sex** |  |  |  |  |  |
| Female | 15302 (54.3%) | 5981 (59.4%) | 3504 (64.8%) | 2795 (40.7%) | 3022 (51.6%) |
| Male | 12904 (45.7%) | 4090 (40.6%) | 1901 (35.2%) | 4077 (59.3%) | 2836 (48.4%) |
| **Race** |  |  |  |  |  |
| White | 26435 (93.7%) | 9679 (96.1%) | 4958 (91.7%) | 6534 (95.1%) | 5264 (89.9%) |
| Black | 572 (2.0%) | 116 (1.2%) | 145 (2.7%) | 112 (1.6%) | 199 (3.4%) |
| Aisan | 605 (2.1%) | 108 (1.1%) | 130 (2.4%) | 123 (1.8%) | 244 (4.2%) |
| Other | 594 (2.1%) | 168 (1.7%) | 172 (3.2%) | 103 (1.5%) | 151 (2.6%) |
| **Smoking status** |  |  |  |  |  |
| Never | 15660 (55.5%) | 5942 (59.0%) | 3093 (57.2%) | 3606 (52.5%) | 3019 (51.5%) |
| Previous | 3012 (10.7%) | 888 (8.8%) | 653 (12.1%) | 680 (9.9%) | 791 (13.5%) |
| Current | 9456 (33.5%) | 3216 (31.9%) | 1646 (30.5%) | 2566 (37.3%) | 2028 (34.6%) |
| Missing | 78 (0.3%) | 25 (0.2%) | 13 (0.2%) | 20 (0.3%) | 20 (0.3%) |
| **Drinking status** |  |  |  |  |  |
| Never | 1217 (4.3%) | 283 (2.8%) | 245 (4.5%) | 244 (3.6%) | 445 (7.6%) |
| Previous | 968 (3.4%) | 231 (2.3%) | 235 (4.3%) | 186 (2.7%) | 316 (5.4%) |
| Current | 26001 (92.2%) | 9551 (94.8%) | 4921 (91.0%) | 6440 (93.7%) | 5089 (86.9%) |
| Missing | 20 (0.1%) | 6 (0.1%) | 4 (0.1%) | 2 (0.0%) | 8 (0.1%) |
| **Dietary score** | 5.06 (1.51) | 4.96 (1.51) | 5.02 (1.54) | 5.13 (1.50) | 5.18 (1.49) |
| **TDI** | -1.29 (3.11) | -1.66 (2.89) | -0.96 (3.27) | -1.59 (2.95) | -0.61 (3.36) |
| **eGFR (mL/min/1.73 m²)** | 95.14 (12.98) | 96.56 (11.82) | 97.00 (12.16) | 93.01 (13.39) | 93.50 (14.49) |
| **ALT (U/L)** | 23.32 (13.97) | 20.22 (11.06) | 20.43 (12.72) | 27.43 (15.61) | 26.48 (15.48) |
| **AST (U/L)** | 26.12 (9.91) | 25.07 (8.84) | 25.08 (10.62) | 27.65 (9.79) | 27.08 (10.73) |
| **CRP (mg/L)** | 2.51 (4.15) | 1.83 (3.66) | 2.47 (4.58) | 2.58 (3.60) | 3.64 (4.82) |

TDI, Townsend deprivation index; eGFR, estimated glomerular filtration rate; ALT, alanine aminotransferase; AST, aspartate aminotransferase; CRP, C-reactive protein; CMH-NF,cardiometabolically healthy and non-frail; CMH-F, cardiometabolically healthy and pre-frail/frail; CMU-NF, cardiometabolically unhealthy and non-frail; CMU-F, cardiometabolically unhealthy and pre-frail/frail; TDI, Townsend deprivation index; eGFR, estimated glomerular filtration rate.

| **Table S20. Cox regression–based mediation analysis of candidate proteomic features linking the cardiometabolic health–frailty phenotype to AF and HF** | | | | | |
| --- | --- | --- | --- | --- | --- |
| **Diseases** | **Proteins** | **Mediation proportions (%)** | **Lower**  **95% CI (%)** | **Upper**  **95% CI (%)** | **P for FDR** |
| AF | ADIPOQ | -47.2 | -74.6 | -19.7 | <0.001 |
| AF | EFNA4 | 26.4 | 11.0 | 41.8 | <0.001 |
| AF | FABP4 | 49.8 | 21.9 | 77.6 | <0.001 |
| AF | UMOD | 19.4 | 7.8 | 30.9 | <0.001 |
| AF | DPT | 25.4 | 9.8 | 41.0 | <0.001 |
| AF | GAL | 15.0 | 5.8 | 24.2 | <0.001 |
| AF | SEZ6L | 13.7 | 5.3 | 22.0 | <0.001 |
| AF | SFRP1 | 18.4 | 6.7 | 30.1 | <0.001 |
| AF | APLP1 | 16.7 | 6.2 | 27.2 | 0.001 |
| AF | TNFRSF10A | 23.7 | 8.2 | 39.2 | 0.002 |
| AF | CA6 | 16.5 | 5.5 | 27.5 | 0.003 |
| AF | FGFBP1 | 15.3 | 4.5 | 26.1 | 0.003 |
| AF | ADA2 | 11.3 | 3.3 | 19.3 | 0.003 |
| AF | CCN5 | 16.7 | 4.7 | 28.7 | 0.006 |
| AF | LGALS4 | 19.5 | 5.2 | 33.8 | 0.006 |
| AF | MEP1A | -18.6 | -32.6 | -4.7 | 0.006 |
| AF | IGFBP1 | -34.6 | -61.6 | -7.7 | 0.007 |
| AF | CTSB | -15.8 | -29.1 | -2.5 | 0.029 |
| AF | HAVCR1 | 14.3 | 2.3 | 26.2 | 0.029 |
| AF | CA14 | 21.2 | 2.6 | 39.9 | 0.045 |
| HF | HAVCR1 | 20.6 | 15.1 | 26.1 | <0.001 |
| HF | EFNA4 | 20.6 | 15.1 | 26.1 | <0.001 |
| HF | FABP4 | 32.9 | 22.7 | 43.1 | <0.001 |
| HF | REN | 11.6 | 7.4 | 15.9 | <0.001 |
| HF | UMOD | 11.9 | 7.4 | 16.5 | <0.001 |
| HF | TNFRSF10A | 16.4 | 10.2 | 22.5 | <0.001 |
| HF | SFRP1 | 11.6 | 7.0 | 16.2 | <0.001 |
| HF | SNCG | 17.6 | 10.7 | 24.4 | <0.001 |
| HF | DPT | 17.1 | 10.2 | 24.0 | <0.001 |
| HF | ADA2 | 8.1 | 4.6 | 11.6 | <0.001 |
| HF | LGALS4 | 14.4 | 8.2 | 20.5 | <0.001 |
| HF | GRN | 8.6 | 4.9 | 12.4 | <0.001 |
| HF | ACE2 | 14.3 | 7.8 | 20.7 | <0.001 |
| HF | VEGFB | 9.5 | 4.6 | 14.3 | <0.001 |
| HF | APLP1 | 8.7 | 4.0 | 13.4 | 0.001 |
| HF | NHLRC3 | 13.8 | 6.3 | 21.3 | 0.002 |
| HF | GSN | 6.2 | 2.6 | 9.9 | 0.003 |
| HF | IL18 | 6.7 | 2.7 | 10.7 | 0.003 |
| HF | CCN5 | 9.2 | 3.7 | 14.7 | 0.004 |
| HF | HYAL1 | 4.3 | 1.4 | 7.3 | 0.011 |
| HF | ADGRG2 | 9.9 | 3.3 | 16.6 | 0.012 |
| HF | FAM20A | 11.0 | 3.6 | 18.5 | 0.012 |
| HF | ADIPOQ | -14.3 | -25.0 | -3.7 | 0.012 |
| HF | CA6 | 7.4 | 2.3 | 12.5 | 0.012 |
| HF | NCS1 | 7.0 | 2.2 | 11.9 | 0.012 |
| HF | CEACAM16 | -5.6 | -9.8 | -1.3 | 0.021 |
| HF | SCARA5 | 8.0 | 2.0 | 14.0 | 0.022 |
| HF | NRCAM | 4.2 | 0.8 | 7.6 | 0.036 |

Mediation analyses were performed using the regmedint package,with CMH-NF as the reference group and CMU-F as the exposed group. Candidate proteins were modeled as continuous mediators using linear mediator models, and HF or AF outcomes were modeled using Cox proportional hazards models.

Models were adjusted for age, sex, race, smoking status, drinking status, dietary score, TDI, eGFR,

HF, heart failure; AF, atrial fibrillation; TDI, Townsend deprivation index; eGFR, estimated glomerular filtration rate.

| **Table S21. Multistate mediation analysis of candidate proteomic features linking the cardiometabolic health–frailty phenotype to AF–HF Comorbidity progression** | | | | | |
| --- | --- | --- | --- | --- | --- |
| **Path** | **Proteins** | **Mediation**  **proportions (%)** | **Lower 95% CI(%)** | **Upper 95% CI(%)** | **P for FDR** |
| Baseline to HF | ACE2 | 15.4 | 8.6 | 22.2 | <0.001 |
| Baseline to HF | ADA2 | 8.2 | 4.5 | 11.9 | <0.001 |
| Baseline to AF | ADA2 | 10.2 | 0.4 | 20.1 | 0.044 |
| Baseline to HF | ADGRG2 | 10.0 | 2.8 | 17.2 | 0.022 |
| Baseline to AF | ADIPOQ | -57.3 | -96.6 | -17.9 | <0.001 |
| Baseline to HF | AGER | 5.2 | 1.5 | 8.9 | 0.020 |
| Baseline to HF | APLP1 | 6.6 | 1.5 | 11.8 | 0.031 |
| Baseline to AF | APLP1 | 16.9 | 3.4 | 30.4 | 0.012 |
| Baseline to HF | CA6 | 8.0 | 2.5 | 13.5 | 0.017 |
| Baseline to AF | CA6 | 14.9 | 1.2 | 28.5 | 0.042 |
| Baseline to HF | CCN5 | 9.1 | 3.2 | 15.1 | 0.011 |
| Baseline to AF | CCN5 | 21.1 | 4.5 | 37.7 | 0.008 |
| Baseline to HF | CEACAM16 | -6.0 | -10.7 | -1.3 | 0.026 |
| Baseline to AF | CTSB | -19.1 | -36.7 | -1.6 | 0.038 |
| Baseline to HF | DPT | 17.4 | 10.0 | 24.8 | <0.001 |
| Baseline to AF | DPT | 30.2 | 8.7 | 51.8 | 0.002 |
| Baseline to HF | EFNA4 | 18.9 | 13.0 | 24.8 | <0.001 |
| Baseline to AF | EFNA4 | 29.6 | 8.4 | 50.8 | <0.001 |
| Baseline to HF | FABP4 | 26.2 | 14.9 | 37.5 | <0.001 |
| Baseline to AF | FABP4 | 56.4 | 18.1 | 94.7 | <0.001 |
| AF to Comorbidity | FABP4 | 59.7 | 14.1 | 105.2 | 0.024 |
| Baseline to HF | FAM20A | 10.6 | 2.6 | 18.6 | 0.029 |
| Baseline to AF | FGFBP1 | 14.7 | 1.1 | 28.4 | 0.034 |
| Baseline to AF | GAL | 19.1 | 6.1 | 32.1 | <0.001 |
| Baseline to HF | GRN | 9.7 | 5.7 | 13.7 | <0.001 |
| Baseline to HF | GSN | 7.6 | 3.7 | 11.5 | <0.001 |
| Baseline to HF | HAVCR1 | 17.5 | 11.8 | 23.2 | <0.001 |
| AF to Comorbidity | HAVCR1 | 49.3 | 14.1 | 84.5 | 0.003 |
| Baseline to AF | IGFBP1 | -36.5 | -71.0 | -1.9 | 0.035 |
| Baseline to HF | IL18 | 6.2 | 1.9 | 10.6 | 0.017 |
| Baseline to HF | LGALS4 | 12.4 | 5.8 | 19.0 | 0.001 |
| Baseline to AF | LGALS4 | 22.2 | 3.0 | 41.3 | 0.022 |
| Baseline to AF | MEP1A | -20.5 | -38.6 | -2.3 | 0.022 |
| Baseline to HF | NHLRC3 | 13.0 | 4.9 | 21.0 | 0.008 |
| Baseline to HF | REN | 10.8 | 6.3 | 15.4 | <0.001 |
| Baseline to AF | SERPINA6 | -13.2 | -25.0 | -1.5 | 0.022 |
| Baseline to AF | SEZ6L | 15.5 | 4.1 | 26.8 | 0.003 |
| Baseline to HF | SFRP1 | 11.1 | 6.0 | 16.2 | <0.001 |
| Baseline to AF | SFRP1 | 23.3 | 6.6 | 39.9 | 0.001 |
| Baseline to HF | SNCG | 14.3 | 6.9 | 21.7 | 0.001 |
| Baseline to HF | TNFRSF10A | 15.5 | 8.8 | 22.2 | <0.001 |
| Baseline to AF | TNFRSF10A | 26.4 | 5.4 | 47.4 | 0.008 |
| Baseline to HF | UMOD | 10.4 | 5.6 | 15.3 | <0.001 |
| Baseline to AF | UMOD | 20.0 | 4.8 | 35.1 | 0.003 |
| Baseline to HF | VEGFB | 9.0 | 3.8 | 14.2 | 0.004 |

Multistate mediation analyses were performed using the regmedint package, with CMH-NF as the reference group and CMU-F as the exposed group. Candidate proteins were modeled as continuous mediators using linear mediator models, and transition-specific endpoints were modeled using Cox proportional hazards models within the multistate framework.

Models were adjusted for age, sex, race, smoking status, drinking status, dietary score, TDI, and eGFR.

HF, heart failure; AF, atrial fibrillation; Comorbidity, AF–HF comorbidity; TDI, Townsend deprivation index; eGFR, estimated glomerular filtration rate.

| **Table S22. Cox regression–based mediation analysis of candidate proteomic features linking the cardiometabolic health–frailty phenotype to AF and HF using mean-imputed proteomic data.** | | | | | |
| --- | --- | --- | --- | --- | --- |
| **Diseases** | **Proteins** | **Mediation proportions (%)** | **Lower 95% CI (%)** | **Upper 95% CI (%)** | **P for FDR** |
| AF | ADIPOQ | -47.1 | -74.4 | -19.8 | <0.001 |
| AF | EFNA4 | 24.8 | 10.1 | 39.6 | <0.001 |
| AF | UMOD | 19.3 | 7.8 | 30.8 | <0.001 |
| AF | FABP4 | 48.9 | 21.3 | 76.5 | <0.001 |
| AF | GAL | 15.0 | 5.9 | 24.2 | <0.001 |
| AF | DPT | 25.0 | 9.5 | 40.4 | <0.001 |
| AF | SFRP1 | 17.5 | 6.1 | 29.0 | 0.002 |
| AF | TNFRSF10A | 22.3 | 7.3 | 37.4 | 0.003 |
| AF | CA6 | 16.6 | 5.7 | 27.5 | 0.003 |
| AF | FGFBP1 | 15.0 | 4.3 | 25.6 | 0.003 |
| AF | ADA2 | 11.1 | 3.2 | 19.0 | 0.003 |
| AF | MEP1A | -19.0 | -33.0 | -5.0 | 0.005 |
| AF | LGALS4 | 19.3 | 5.2 | 33.4 | 0.006 |
| AF | IGFBP1 | -34.6 | -61.6 | -7.7 | 0.008 |
| AF | CCN5 | 15.5 | 3.8 | 27.1 | 0.011 |
| AF | CTSB | -15.6 | -28.9 | -2.3 | 0.032 |
| AF | HAVCR1 | 14.0 | 2.2 | 25.9 | 0.032 |
| AF | CA14 | 20.9 | 2.4 | 39.5 | 0.049 |
| HF | EFNA4 | 20.0 | 14.6 | 25.3 | <0.001 |
| HF | HAVCR1 | 20.3 | 14.9 | 25.8 | <0.001 |
| HF | FABP4 | 32.3 | 22.1 | 42.5 | <0.001 |
| HF | REN | 11.4 | 7.2 | 15.6 | <0.001 |
| HF | TNFRSF10A | 16.2 | 10.2 | 22.2 | <0.001 |
| HF | UMOD | 11.9 | 7.4 | 16.5 | <0.001 |
| HF | SFRP1 | 11.5 | 7.0 | 16.0 | <0.001 |
| HF | PI3 | 6.9 | 4.1 | 9.8 | <0.001 |
| HF | DPT | 16.4 | 9.5 | 23.2 | <0.001 |
| HF | ADA2 | 7.8 | 4.4 | 11.2 | <0.001 |
| HF | LGALS4 | 14.2 | 8.1 | 20.3 | <0.001 |
| HF | ACE2 | 14.2 | 8.0 | 20.5 | <0.001 |
| HF | NHLRC3 | 13.4 | 6.2 | 20.7 | 0.002 |
| HF | GSN | 6.1 | 2.5 | 9.8 | 0.004 |
| HF | CCN5 | 9.3 | 3.8 | 14.7 | 0.004 |
| HF | HYAL1 | 4.2 | 1.3 | 7.1 | 0.013 |
| HF | FAM20A | 10.9 | 3.5 | 18.3 | 0.013 |
| HF | ADGRG2 | 9.8 | 3.2 | 16.4 | 0.013 |
| HF | NCS1 | 7.0 | 2.2 | 11.8 | 0.013 |
| HF | CA6 | 7.3 | 2.3 | 12.4 | 0.014 |
| HF | SCARA5 | 8.1 | 2.2 | 14.1 | 0.019 |
| HF | ADIPOQ | -13.5 | -24.0 | -3.0 | 0.019 |
| HF | CEACAM16 | -5.6 | -9.9 | -1.3 | 0.021 |
| HF | NRCAM | 4.4 | 0.9 | 7.8 | 0.031 |

Mediation analyses were performed using the regmedint package,with CMH-NF as the reference group and CMU-F as the exposed group. Candidate proteins were modeled as continuous mediators using linear mediator models, and HF or AF outcomes were modeled using Cox proportional hazards models.

Models were adjusted for age, sex, race, smoking status, drinking status, dietary score, TDI, eGFR,

HF, heart failure; AF, atrial fibrillation; TDI, Townsend deprivation index; eGFR, estimated glomerular filtration rate.

| **Table S23. Multistate mediation analysis of candidate proteomic features linking the cardiometabolic health–frailty phenotype to AF–HF comorbidity progression using mean-imputed proteomic data.** | | | | | |
| --- | --- | --- | --- | --- | --- |
| **Path** | **Proteins** | **Mediation  proportions (%)** | **Lower 95% CI(%)** | **Upper 95% CI(%)** | **P for FDR** |
| Baseline to HF | ACE2 | 15.3 | 8.7 | 21.9 | <0.001 |
| Baseline to HF | ADA2 | 7.9 | 4.3 | 11.6 | <0.001 |
| Baseline to AF | ADA2 | 10.2 | 0.4 | 20.0 | 0.040 |
| Baseline to HF | ADGRG2 | 10.0 | 2.9 | 17.2 | 0.022 |
| Baseline to AF | ADIPOQ | -57.2 | -96.5 | -18.0 | <0.001 |
| Baseline to HF | AGER | 5.0 | 1.3 | 8.7 | 0.025 |
| Baseline to HF | CA6 | 7.9 | 2.5 | 13.4 | 0.017 |
| Baseline to AF | CA6 | 15.1 | 1.5 | 28.8 | 0.040 |
| Baseline to HF | CCN5 | 9.1 | 3.3 | 15.0 | 0.010 |
| Baseline to AF | CCN5 | 19.5 | 3.5 | 35.5 | 0.017 |
| Baseline to HF | CEACAM16 | -5.9 | -10.6 | -1.3 | 0.029 |
| Baseline to AF | CTSB | -18.9 | -36.4 | -1.4 | 0.040 |
| Baseline to HF | DPT | 16.4 | 9.0 | 23.7 | <0.001 |
| Baseline to AF | DPT | 29.5 | 8.2 | 50.7 | 0.002 |
| Baseline to HF | EFNA4 | 17.9 | 12.2 | 23.6 | <0.001 |
| Baseline to AF | EFNA4 | 27.6 | 7.4 | 47.7 | 0.002 |
| AF to Comorbidity | EFNA4 | 30.7 | 7.1 | 54.3 | 0.020 |
| Baseline to HF | FABP4 | 25.4 | 14.1 | 36.7 | <0.001 |
| Baseline to AF | FABP4 | 55.1 | 17.3 | 92.9 | 0.001 |
| AF to Comorbidity | FABP4 | 60.2 | 14.5 | 105.9 | 0.019 |
| Baseline to HF | FAM20A | 10.4 | 2.4 | 18.4 | 0.034 |
| Baseline to AF | FGFBP1 | 14.5 | 1.0 | 28.1 | 0.036 |
| Baseline to AF | GAL | 19.1 | 6.1 | 32.1 | 0.001 |
| Baseline to HF | GSN | 7.7 | 3.8 | 11.6 | <0.001 |
| Baseline to HF | HAVCR1 | 17.4 | 11.7 | 23.0 | <0.001 |
| AF to Comorbidity | HAVCR1 | 48.9 | 13.8 | 83.9 | 0.003 |
| Baseline to AF | IGFBP1 | -36.4 | -71.0 | -1.9 | 0.036 |
| Baseline to HF | LGALS4 | 12.2 | 5.7 | 18.8 | 0.001 |
| Baseline to AF | LGALS4 | 22.1 | 3.1 | 41.2 | 0.020 |
| Baseline to AF | MEP1A | -20.9 | -39.2 | -2.6 | 0.020 |
| Baseline to HF | NHLRC3 | 12.8 | 5.0 | 20.6 | 0.007 |
| Baseline to HF | PI3 | 5.7 | 2.6 | 8.8 | 0.001 |
| Baseline to HF | REN | 10.6 | 6.1 | 15.0 | <0.001 |
| Baseline to AF | SERPINA6 | -12.7 | -24.3 | -1.2 | 0.027 |
| Baseline to HF | SFRP1 | 10.9 | 5.8 | 15.9 | <0.001 |
| Baseline to AF | SFRP1 | 22.0 | 5.9 | 38.2 | 0.002 |
| Baseline to HF | TNFRSF10A | 15.0 | 8.4 | 21.6 | <0.001 |
| Baseline to AF | TNFRSF10A | 24.5 | 4.3 | 44.8 | 0.017 |
| Baseline to HF | UMOD | 10.4 | 5.6 | 15.3 | <0.001 |
| Baseline to AF | UMOD | 19.9 | 4.8 | 35.0 | 0.003 |

Multistate mediation analyses were performed using the regmedint package, with CMH-NF as the reference group and CMU-F as the exposed group. Candidate proteins were modeled as continuous mediators using linear mediator models, and transition-specific endpoints were modeled using Cox proportional hazards models within the multistate framework.

Models were adjusted for age, sex, race, smoking status, drinking status, dietary score, TDI, and eGFR.

HF, heart failure; AF, atrial fibrillation; Comorbidity, AF–HF comorbidity; TDI, Townsend deprivation index; eGFR, estimated glomerular filtration rate.

| **Table S24. Cox regression–based mediation analysis of candidate proteomic features linking the cardiometabolic health–frailty phenotype to AF and HF using complete case proteomic data.** | | | | | |
| --- | --- | --- | --- | --- | --- |
| **Diseases** | **Proteins** | **Mediation proportions (%)** | **Lower 95% CI (%)** | **Upper 95% CI (%)** | **P for FDR** |
| HF | FABP4 | 31.6 | 20.9 | 42.2 | <0.001 |
| AF | FABP4 | 44.6 | 18.5 | 70.8 | <0.001 |

Mediation analyses were performed using the regmedint package,with CMH-NF as the reference group and CMU-F as the exposed group. Candidate proteins were modeled as continuous mediators using linear mediator models, and HF or AF outcomes were modeled using Cox proportional hazards models.

Models were adjusted for age, sex, race, smoking status, drinking status, dietary score, TDI, eGFR,

HF, heart failure; AF, atrial fibrillation; TDI, Townsend deprivation index; eGFR, estimated glomerular filtration rate.

| **Table S25. Multistate mediation analysis of candidate proteomic features linking the cardiometabolic health–frailty phenotype to AF–HF comorbidity progression using complete case proteomic data.** | | | | | |
| --- | --- | --- | --- | --- | --- |
| **Path** | **Proteins** | **Mediation  proportions (%)** | **Lower 95% CI(%)** | **Upper 95% CI(%)** | **P for FDR** |
| Baseline to HF | FABP4 | 23.7 | 11.7 | 35.7 | 0.001 |
| Baseline to HF | CNTN5 | 6.9 | 1.4 | 12.4 | 0.031 |
| Baseline to AF | FABP4 | 48.8 | 14.1 | 83.4 | 0.002 |
| AF to com | FABP4 | 62.9 | 14.8 | 111.0 | 0.002 |

Multistate mediation analyses were performed using the regmedint package, with CMH-NF as the reference group and CMU-F as the exposed group. Candidate proteins were modeled as continuous mediators using linear mediator models, and transition-specific endpoints were modeled using Cox proportional hazards models within the multistate framework.

Models were adjusted for age, sex, race, smoking status, drinking status, dietary score, TDI, and eGFR.

HF, heart failure; AF, atrial fibrillation; Comorbidity, AF–HF comorbidity; TDI, Townsend deprivation index; eGFR, estimated glomerular filtration rate.

**Figure S1. The study flow chart.**
**Figure S2. AF–HF multistate disease transition framework.**
The figure illustrates the prespecified multistate framework used to characterize AF–HF disease progression among 319,048 participants initially free of AF and HF. Transition A denotes progression from baseline to incident HF; transition B, from baseline to incident AF; transition C, from HF to comorbidity; and transition D, from AF to comorbidity. During follow-up, 5,410 participants developed incident HF, 17,844 developed incident AF, 791 progressed from HF to comorbidity, and 2,403 progressed from AF to comorbidity, yielding 3,194 participants with comorbidity. Percentages indicate the proportion of participants experiencing each transition from the corresponding source state.

AF, atrial fibrillation; HF, heart failure.

**Figure S3. Model-adjusted cumulative transition probabilities for AF–HF disease progression according to cardiometabolic health–frailty phenotype**.

Model-adjusted cumulative transition probabilities are shown across follow-up for four disease-transition pathways: baseline to HF, baseline to AF, HF to comorbidity, and AF to comorbidity. Estimates are presented according to cardiometabolic health–frailty phenotype: CMH-NF, CMH-F, CMU-NF, and CMU-F.

Models were adjusted for age, sex, race, smoking status, drinking status, dietary score, TDI, eGFR.

AF, atrial fibrillation; HF, heart failure; CMH-F, cardiometabolically healthy and pre-frail/frail; CMH-NF, cardiometabolically healthy and non-frail; CMU-F, cardiometabolically unhealthy and pre-frail/frail; CMU-NF, cardiometabolically unhealthy and non-frail; TDI, Townsend deprivation index; eGFR, estimated glomerular filtration rate.

**Figure S4. Subgroup associations of cardiometabolic health–frailty phenotypes with AF–HF transitions by age and sex.**

Forest plots show the hazard ratios (HRs) and 95% confidence intervals (CIs) for the associations of cardiometabolic health–frailty phenotypes with four transitions: from baseline to HF, from baseline to AF, from HF to comorbidity, and from AF to comorbidity, stratified by age and sex. HRs and 95% CIs were derived from multivariable multistate models, with CMH-NF participants as the reference group.

Models were adjusted for age, sex, race, smoking status, drinking status, dietary score, TDI, and eGFR, as appropriate, excluding the corresponding stratification variable. P values for interaction were calculated to assess heterogeneity of associations across age and sex subgroups, with false discovery rate correction applied for multiple testing.

HF, heart failure; AF, atrial fibrillation; Comorbidity, AF–HF comorbidity; CMH-NF, cardiometabolically healthy and non-frail; CMH-F, cardiometabolically healthy and pre-frail/frail; CMU-NF, cardiometabolically unhealthy and non-frail; CMU-F, cardiometabolically unhealthy and pre-frail/frail; TDI, Townsend deprivation index; eGFR, estimated glomerular filtration rate.

**Figure S5. Model selection, phenotype distribution, and internal reproducibility of the three-class cardiometabolic–frailty solution**

(A) Model-selection diagnostics in the training set. The left plot presents the Akaike information criterion (AIC), Bayesian information criterion (BIC), sample-size-adjusted BIC (aBIC), and consistent AIC (CAIC) for models with one to six latent classes; lower values indicate better relative fit. The middle plot presents entropy and the minimum and mean class-specific average posterior probabilities (AvePP) for models with two to six classes; higher values indicate clearer class separation. The horizontal dotted line at 0.70 is a heuristic reference for AvePP only and is not a threshold for entropy. The right plot presents the smallest class proportions based on model-estimated membership probabilities and modal assignment, whereby each participant was assigned to the class with the highest posterior probability. The horizontal dotted line at 5% identifies potentially small or unstable classes. These reference values were used as supportive diagnostic criteria rather than mandatory selection thresholds. Classification diagnostics are not applicable to the one-class model. The vertical dashed line indicates the selected three-class solution, chosen through an integrated assessment of relative model fit, classification quality, class size, parsimony, clinical interpretability, and reproducibility in the validation set.

(B) Distribution of the three latent cardiometabolic–frailty phenotypes in the training set (n=191,429) and validation set (n=127,619). Sector labels indicate the proportions assigned to each class, while the legends report the corresponding numbers and percentages. Class membership was determined by modal assignment from the selected three-class solution.

(C) Heatmaps of class-specific conditional response probabilities for the selected three-class solution in the training and validation sets. Rows represent five binary cardiometabolic indicators and the pre-frail and frail categories of the three-category frailty indicator, and columns represent Classes 1–3. Values within cells are percentages, with darker shading indicating higher probabilities.

(D) Radar plots comparing the class-specific conditional response probability profiles between the training and validation sets. Columns represent Classes 1–3, and rows represent the training and validation sets. The radial scale ranges from 0% to 100%, with values farther from the centre indicating higher conditional response probabilities.

In Panels C and D, robust status is not displayed because its conditional probability is complementary to those of pre-frail and frail status. Adiposity denotes overweight/obesity or central adiposity. AvePP, average posterior probability; HDL-C, high-density lipoprotein cholesterol.

**Figure S6. Plasma proteomic signatures associated with cardiometabolic health and frailty and pathway enrichment with feature selection stability.**

A. PWAS of cardiometabolic health and frailty status with circulating proteins. The x-axis shows the PWAS β coefficient for the association of cardiometabolic health status or frailty status with circulating protein abundance, and the y-axis shows statistical significance expressed as −log10 P values. Multivariable-adjusted plasma PWAS were conducted separately for cardiometabolic health status and frailty status across 2,911 circulating proteins. A two-sided P value <0.05/2911 was considered proteome-wide significant after Bonferroni correction. Models were adjusted for age, sex, race, smoking status, drinking status, TDI, dietary score, and eGFR. Grey points indicate proteins that did not reach proteome-wide significance, pink points indicate PWAS-significant proteins only, and bright red points indicate PWAS-significant proteins retained by elastic-net feature selection. IL17RB, interleukin-17 receptor B; CTSB, cathepsin B; CLUL1, clusterin-like protein 1; WFIKKN2, WAP, Kazal, immunoglobulin, Kunitz and NTR domain-containing protein 2; MENT, myeloid and erythroid nuclear termination stage-specific protein; FGFR2, fibroblast growth factor receptor 2; HEG1, heart development protein with EGF-like domains 1; GOLM2, Golgi membrane protein 2; PTH, parathyroid hormone; SIT1, signalling threshold-regulating transmembrane adaptor 1; ADAMTS8, a disintegrin and metalloproteinase with thrombospondin motifs 8; ITGAV, integrin αV; NTRK3, neurotrophic receptor tyrosine kinase 3; ADGRG2, adhesion G protein-coupled receptor G2; CA14, carbonic anhydrase 14; FABP4, fatty acid-binding protein 4; ADM, adrenomedullin; GDF15, growth differentiation factor 15; TNFRSF11A, tumour necrosis factor receptor superfamily member 11A; IGFBP4, insulin-like growth factor-binding protein 4.

B. Functional enrichment analysis of proteins associated with cardiometabolic health and frailty.

Bubble plots illustrate GO, KEGG, and Reactome pathway enrichment results for proteins associated with cardiometabolic health and frailty.

Panels I–II show enrichment results for cardiometabolic health-associated proteins, including GO BP, CC, and MF terms (I), as well as KEGG and Reactome pathways (II). Panels III–IV present corresponding analyses for frailty-associated proteins. The x-axis represents the GeneRatio, and bubble size reflects the number of proteins mapped to each term or pathway. Bubble color indicates the FDR-adjusted P value. Enrichment significance was defined as FDR-adjusted P < 0.05.

GO, Gene Ontology; KEGG, Kyoto Encyclopedia of Genes and Genomes; BP, biological process; CC, cellular component; MF, molecular function

C. Stability of protein selection across elastic-net regression models. Venn diagrams illustrate the overlap of proteins selected by elastic-net models across four regularization mixing parameters (α = 0.25, 0.50, 0.75, and 1.00). Numbers indicate proteins uniquely or jointly selected under different α values. Proteins consistently selected across all four α values were defined as stable proteins. These stable proteins shared between cardiometabolic health and frailty were carried forward as candidate mediators in downstream mediation analyses.
